# Development of a halofluorocarbon, chromatography-free radiosynthesis of fluorine-18 difluorocarbene

**DOI:** 10.1186/s41181-025-00353-8

**Published:** 2025-07-14

**Authors:** Catherine G. F. Dickmann, Andrew D. Bond, Selena Milicevic Sephton, Franklin I. Aigbirhio

**Affiliations:** 1 Molecular Imaging Chemistry Laboratory, Department of Clinical Neurosciences, Wolfson Brain Imaging Centre, University of Cambridge, Cambridge, CB2 0SZ UK; 2 Yusuf Hamied Department of Chemistry, University of Cambridge, Lensfield Road, Cambridge, CB2 1 EW UK

**Keywords:** Radiochemistry, Chemistry, Small-molecules

## Abstract

**Background:**

In recent years, the development of the [^18^F]difluoromethyl radical ([^18^F]2-((difluoromethyl)sulfonyl)benzo[*d*]thiazole, [^18^F]**4**), and [^18^F]difluorocarbene ([^18^F]1-chloro-4-((difluoromethyl)sulfonyl)benzene, [^18^F]**10**) prosthetic groups, has paved the way towards direct ^18^F-difluoromethylation in routine PET tracer synthesis with high molar activity. However, limitations in their syntheses may be hindering their widespread adoption by the radiochemistry community. Firstly, the synthesis of the precursors 2-((bromofluoromethyl)thio)benzo[*d*]thiazole (**3**) and (bromofluoromethyl)(4-chlorophfenyl)sulfane (**8**) requires the use of the ozone-depleting dibromofluoromethane, a reagent that is not-commercially available. Secondly, the reported syntheses of [^18^F]**4** and [^18^F]**10** are lengthy and require semi-preparative HPLC purification prior to the ^18^F-difluoromethylation step. Finally, in the case of [^18^F]**10**, very large amounts of precursor material (200 μmol) are required for difluorocarbene insertion. The aim of this work was to develop a halofluorocarbon-free radiosynthesis of [^18^F]**4** and [^18^F]**10** on the GE TRACERlab FX_FN_ module. Additionally, another aim was to develop a chromatography-free, fully-automated synthesis of [^18^F]**10** on the GE FX_FN_ module.

**Results:**

Precursors **3** and **8** were synthesised in 21% and 54% yield via decarboxylative bromination, which circumvented the need for ozone-depleting dibromofluoromethane. Difluoromethyl reagents [^18^F]**4** and [^18^F]**10** were synthesised on a GE FX_FN_ module with semi-prep HPLC purification in 4% and 3% RCY (decay-corrected), respectively. The synthesis of [^18^F]**10** was further simplified through elimination of the semi-prep HPLC purification in favour of a cartridge-based solid-phase extraction (SPE) trapping and elution approach (on an alumina SPE cartridge loaded in series with a C18 Sep-Pak plus SPE cartridge) to give [^18^F]**10** in 10.1% ± 1.9% (*n* = 6, decay-corrected) RCY (97% ± 3% RCP, 1.5–11 GBq/μmol). Finally, a fully automated ^18^F-difluoromethylation radiosynthesis with [^18^F]**10** was developed on two GE FX_FN_ modules linked together to yield the model ^18^F-difluoromethylated compound in adequate amounts for biological studies, in under two hours (99.0 MBq, 0.8% RCY {decay-corrected}, 1.5 GBq/μmol, 103 min total synthesis time). Therefore, we have established a path forward for routine automated synthesis of radiotracers via [^18^F]difluorocarbene insertion with [^18^F]**10**.

**Conclusions:**

A halofluorocarbon, chromatography-free synthesis on the GE FX_FN_ module afforded difluorocarbene reagent [^18^F]**10** in 10.1% ± 1.9% RCY (decay-corrected). Additionally, a fully-automated three-step [^18^F]difluorocarbene insertion radiosynthesis using two tandem FX_FN_s is described for the first time, providing a path forward to the full automation of [^18^F]difluorocarbene insertion on two-reactor systems.

**Graphical abstract:**

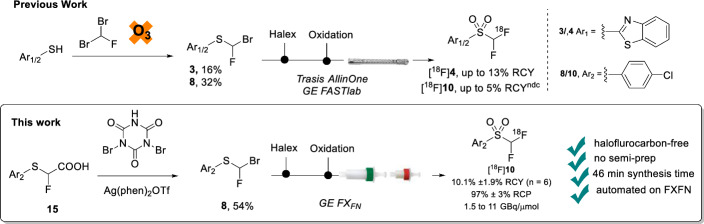

**Supplementary Information:**

The online version contains supplementary material available at 10.1186/s41181-025-00353-8.

## Background

Driven by recent advances in fluorine-18 difluoromethyl radiochemical methodology, the [^18^F]difluoromethyl group has garnered interest within the PET radiotracer development community (Ford et al. 2024; Mishra et al. 2025; Ortalli et al. 2024). In a medicinal chemistry context, the non-labelled difluoromethyl group possesses unique physiochemical properties which can greatly enhance the physiological activities of difluoromethyl-containing drug compounds (Meanwell 2018; Sap et al. 2021; Xing et al. 2015). For instance, the difluoromethyl group is less lipophilic than the trifluoromethyl group, can act as a hydrogen bond donor, modulates drug conformation, and is a bioisostere of the hydroxyl, thiol or methyl groups (Mao et al. 2024; Zafrani et al. 2017). The difluoromethyl group can also impart metabolic stability, for example the non-labelled difluoromethyl group has been shown to block metabolism by aldehyde oxidase (O’Hara et al. 2014). Thus, there is a strong impetus from a radiopharmaceutical development standpoint to develop robust and accessible protocols for incorporating [^18^F]difluoromethyl groups into small molecules given their ubiquity in drug development. This reflects a broader interest across medicinal chemistry in incorporating the non-labelled difluoromethyl group into drug-like molecules (Meanwell 2018).

**Fig. 1:**
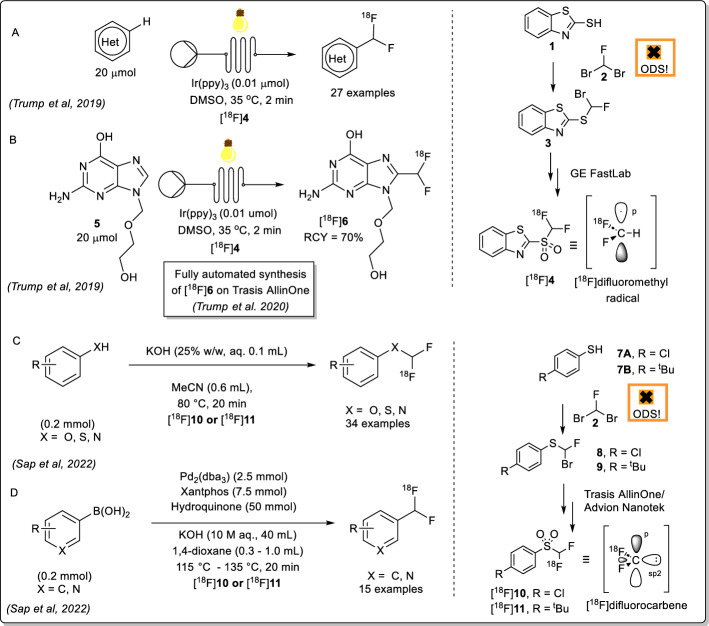
Direct ^18^F-difluoromethylation with [^18^F]difluoromethyl radical and [^18^F]difluorocarbene. ODS, ozone-depleting substance

In the past 5 years, development of [^18^F]difluorocarbene and [^18^F]difluoromethyl radical prosthetic groups has paved the way towards direct ^18^F-difluoromethylation in routine PET tracer synthesis with high molar activity (Fig. 1). These ^18^F-difluoromethylation reagents take inspiration from sulfone-containing fluorinating reagents which are employed in traditional synthetic chemistry, particularly those developed by the group of Jinbo Hu (Ni et al. 2015; Sap et al., Xie & Hu 2024).

The first example of direct radical ^18^F-difluoromethylation with the sulfone reagent [^18^F]**4** (Fig. 1A) was reported in 2019 (Trump et al. 2019) where [^18^F]**4** was used to ^18^F-difluoromethylate a range of (N)-heteroaromatics, including acyclovir (**5**), under iridium photocatalytic conditions in moderate to high yields (e.g. [^18^F]**6** in 70% RCY).^1^ [^18^F]**4** was radiosynthesised in up to 13% RCY on the GE FastLab module (Trump et al. 2019) from a bromofluoromethane precursor **3**, which is obtained in-turn from nucleophilic substitution of **1** with dibromofluoromethane (**2**). However, this reagent (**2**) is no longer commercially available due to its ozone-depleting properties (UNEP 2000). This three-step ^18^F-difluoromethylation process has also been fully automated for the synthesis of [^18^F]**6** on the Trasis AllinOne module with an in-built flow reactor (two-step [^18^F]**4** synthesis and one-step photocatalytic ^18^F-difluoromethylation of **5**) (Trump et al. 2020, Fig. 1B).

Following this report, in 2022 two sulfone [^18^F]difluorocarbene reagents, [^18^F]**10** and [^18^F]**11** (Fig. 1A, B) and their use for difluorocarbene-mediated difluoromethylation were described (Sap et al. 2022). These fluorine-18 difluorocarbene reagents were radiosynthesised on the Advion Nanotek and Trasis AllinOne modules in up to 5% RCY^ndc^ from precursors **8** or **9**—similarly obtained from nucleophilic substitution of **7A** or **7B** with ozone-depleting dibromofluromethane (**2**) (Sap et al. 2022). Strikingly, [^18^F]**10** and [^18^F]**11** underwent both non-automated X–H insertion reactions (34 examples) as well as Pd-catalysed ^18^F-difluoromethylations from boronic acid precursors (15 examples) with moderate to high radiochemical conversions (Fig. 1C, D). However, both insertion and Pd-catalysed ^18^F-difluoromethylations from [^18^F]difluorocarbene, required large amounts of precursor materials (0.2 mmol) which is normally incompatible with automated radiosynthesis Moreover, a fully automated three-step [^18^F]difluorocarbene-mediated ^18^F-difluoromethylation sequence has not yet been realised for either of these processes.

Therefore, the overarching aim of this present study was to translate the radiosynthesis of these ^18^F-difluoromethylating reagents to non-cassette based radiosynthesis modules such as the GE TRACERlab FX_FN_ module, thereby widening the utility of this methodology. Furthermore, specific emphasis was placed on exploring reaction parameters surrounding [^18^F]diflurocarbene insertions owing to a parallel interest in our lab to develop PET radiotracers via [^18^F]difluorocarbene insertion. Difluorocarbene reagent [^18^F]**10** was selected for this optimisation.

Specifically the aims of this study were to: (i) develop an alternative halofluorocarbon-free synthesis of precursors **3** and **8**, (ii) develop a radiosynthesis of [^18^F]**4** and [^18^F]**10** on the GE FX_FN_ module, (iii) develop a chromatography-free synthesis of [^18^F]**10** on the GE FX_FN_ module, (iv) explore [^18^F]difluorocarbene insertion efficiency with [^18^F]**10** as a function of precursor and base amount, (v) develop a fully automated three-step [^18^F]difluorocarbene insertion radiosynthesis using two tandem GE FX_FN_ modules.

## Results

### Syntheses of precursors 3 and 8 via Ag(I) catalysed-decarboxylative bromination

**Fig. 2:**
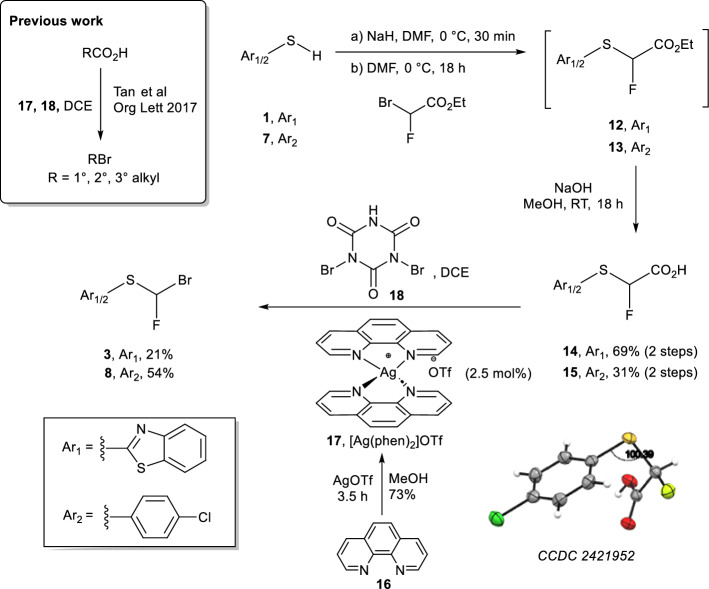
Synthesis of [^18^F]difluoromethylating reagent precursors via decarboxylative bromination

The required catalyst Ag(phen)_2_OTf (**17**) was synthesised in 73% yield (Figure 2). 2-Mercaptobenzothiazole (**1**) and 4-chlorothiophenol (**7**) were alkylated with ethylbromofluoroacetate under basic conditions to yield intermediate ethylfluoroesters **12** and **13**, respectively. Assuming full conversion, these intermediate esters were saponified to give the desired α-fluoroacids **14** and **15** in 69% and 31% yields respectively (over two-steps) and the structure of **15** was further confirmed by X-ray crystallography (Figure 2, CCDC 2421952).

Using Tan and co-workers’ modified conditions to acid **14**, desired bromofluorosulfide precursor **3** was obtained at 70 °C in 21% yield (Supplementary Fig. 2). Bromination of 4-(chloro)phenyl acid **15** was performed at 0 °C to afford **8** in up to 54% yield.

### Syntheses and characterisation of unlabelled 4 and 10

**Fig. 3:**
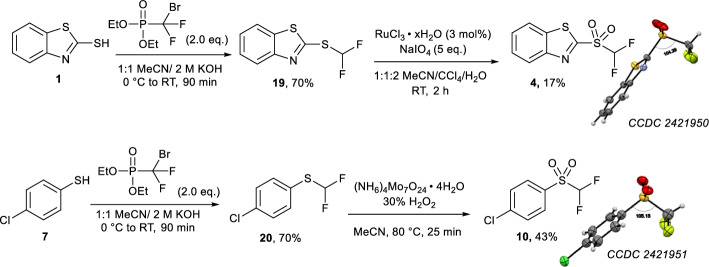
Synthesis of non-radioactive reference compounds **4** and **10**, with X-ray crystallography structures at right

Non-radioactive reference compounds **4** and **10** were synthesised from 2-mercaptobenzothiazole (**1**) and 4-(chloro)thiophenol (**7**), respectively (Fig. 3, CCDC 2421952, CCDC 2421951, respectively). 2-Mercaptobenzathiazole (**1**) was alkylated using diethyl(bromodifluoromethyl)phosphonate under aqueous basic conditions to yield sulfide **19** in 70% yield, which underwent subsequent RuCl_3_ xH_2_O/NaIO_4_ oxidation to yield reference difluorocarbene **4** in 17% yield. In a similar process, sulfide **20** was obtained by alkylation of 4-(chloro)thiophenol **7** in 70% yield. Sulfide **20** was subsequently oxidized with (NH_4_)_6_Mo_7_O_24_ · 4H_2_O/30% H_2_O_2_ to yield **10** in 43% yield. The structures of **4** and **10** were further confirmed by X-ray crystallography and deposited at the CCDC (Fig. 3), adding to the body of structural information around organic difluoromethylating reagents.

### Radiosynthesis of [^18^F]4 and [^18^F]10 on the GE FXFN module

**Fig. 4:**
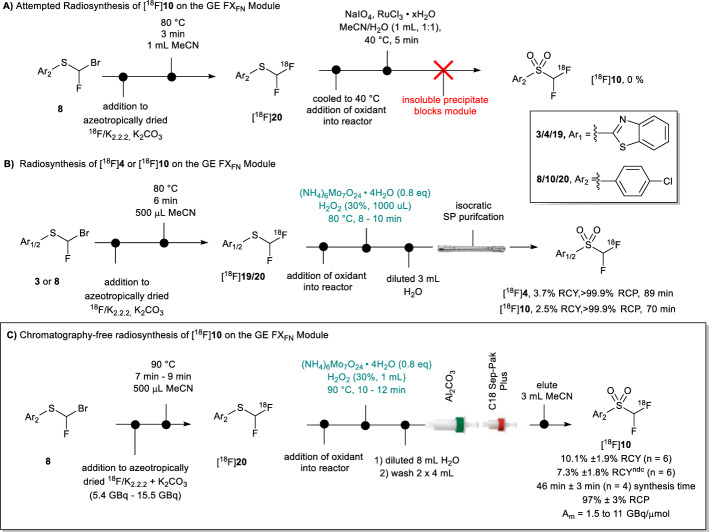
Synthesis of [^18^F]**4** and [^18^F]10 on the GE FX_FN_. **A** Failed synthesis of [^18^F]**10** on the GE FX_FN_ module with ruthenium chloride/periodate oxidation system. **B** Radiosynthesis of [^18^F]**4** and [^18^F]**10** on GE FX_FN_ module with semi-prep purification. (**C**) Chromatography-free radiosynthesis of [^18^F]**10** on the GE FX_FN_ module

[^18^F]**4** was successfully radiosynthesised from **3** (11.4 mg) on the GE FX_FN_ using a Halex reaction at 90 °C for 6 min followed by oxidation with the (NH_6_)_4_Mo_7_O_24_ · 4H_2_O – peroxide oxidant system for 8–10 min at 80 °C. Radiolabelled product was isolated in greater than 99% RCP in 3.7% RCY after semi-preparative purification (Fig. 6B, Supplementary Fig. 3). Similarly, we applied these conditions to the radiosynthesis of fluorine-18 difluorocarbene reagent [^18^F]**10** from **8** (13.2 mg) (Fig. 4B, Supplementary Fig. 4), which was isolated in greater than 99% RCP and RCY of 2.5%, which is within range of the yields reported by Sap and co-workers for [^18^F]**10** (between 1 and 5% RCY).

### Chromatography-free radiosynthesis of [^18^F]10 on the GE FXFN module

**Fig. 5:**
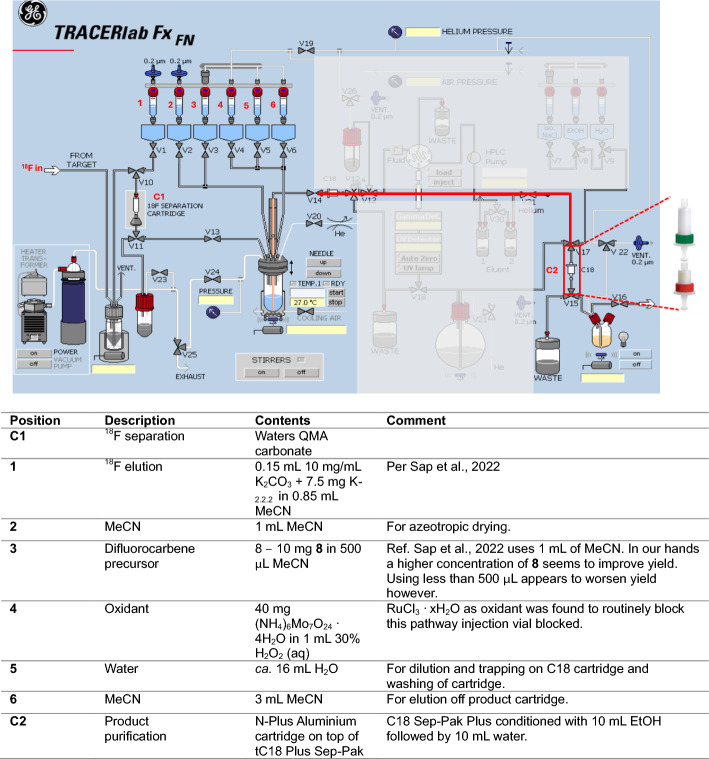
Annotated schematic of GE FX_FN_ module set-up for chromatography-free synthesis of [^18^F]**10**. A key connection between reactor vessel and C2 is highlighted in red showing where the alumina and C18 purification cartridge are fitted. Areas which are not necessary for this synthesis are greyed out

Figure 5 depicts how the GE FX_FN_ module is prepared for the chromatography-free synthesis of [^18^F]**10.**

The GE FX_FN_ was configured as shown in Fig. 5 such that the internal semi-HPLC system is bypassed, and the reactor feeds directly to the formulation cartridge position (see Supplementary Fig. 5 for photo of this modified set-up). The crude reaction mixture was loaded onto an alumina solid-phase extraction (SPE) cartridge in series with a C18 Plus SPE cartridge which is used to purify [^18^F]**10.** On completion of the oxidation step, the reactor was diluted with water and the product trapped on the C18 SPE cartridge, followed by two aqueous washes through the reactor and finally elution with MeCN into the product vial. Using this chromatography-free procedure [^18^F]**10** could be successfully radiosynthesised in up to 14% RCY, with average RCY of 10.1 ± 1.9% (*n* = 6) and RCP of 97% ± 3%, with molar activities ranging between 1.5 GBq/μmol and 11 GBq/μmol (Supplementary Figs. 6 and 7 and Supplementary Table 2).

### ^18^F-difluoromethylation validation with [^18^F]10 from chromatography-free radiosynthesis

Using [^18^F]**22** it was found that reducing the phenolic precursor amount **21** eightfold still afforded [^18^F]**22** in moderate radiochemical conversions: 38% ± 2% (*n* = 3) radiochemical conversion compared with 72% ± 0% (*n* = 2) obtained from literature conditions (Supplementary Fig. 8).

**Fig. 6:**
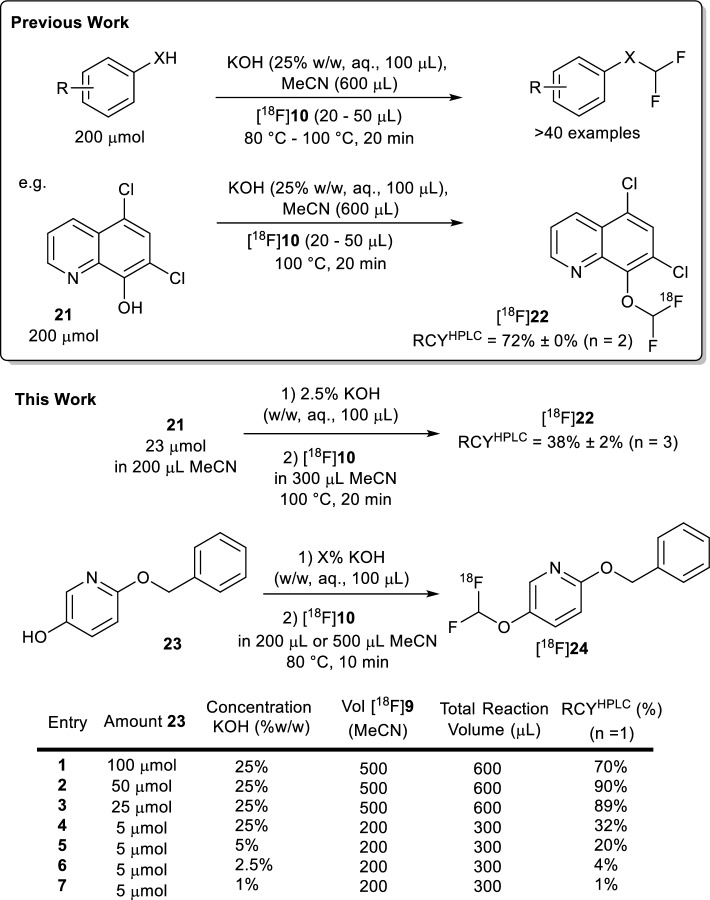
^**1**8^F-difluoromethylation with SPE cartridge-purified [^18^F]10 with two model systems **22** and **24**. RCY.^HPLC^ refers HPLC-determined radiochemical yield (product not isolated). See Supplementary Fig. 10 for synthesis of non-labelled reference compounds **22** and **24**

With compound [^18^F]**24**, two, four and eight-fold reductions in precursor (**23**) amount from literature conditions (200 μmol precursor, 0.1 ml 25% w/w KOH, 600 μL reaction volume) were tolerated, yielding high radiochemical conversions (RCY^HPLC^ = 70%, 90%, 89%, respectively) (Fig. 6, Supplementary Fig. 7). A 40-fold reduction to 5 μmol of precursor reduced the radiochemical conversion to a moderate 32%. Reducing the total reaction volume to 300 μL and reducing the base concentration (5%, 2.5% and 1% w/w KOH) further reduced radiochemical conversions for 5 μmol of precursor (20%, 4% and 1%, respectively).

### Synthesis of [^18^F]24 using fully-automated three-step procedure

Full automation was achieved by using two GE FX_FN_s in adjacent hot cells which were configured such that the [^18^F]**10** synthesis was completed on one GE FX_FN_ and the product [^18^F]**10** dispensed into the reactor of a second neighbouring GE FX_FN_ where the [^18^F]difluorocarbene insertion reaction could be automated and product purified by semi-prep purification (see Supplementary Fig. 11 for schematic of set-up).

**Fig. 7:**
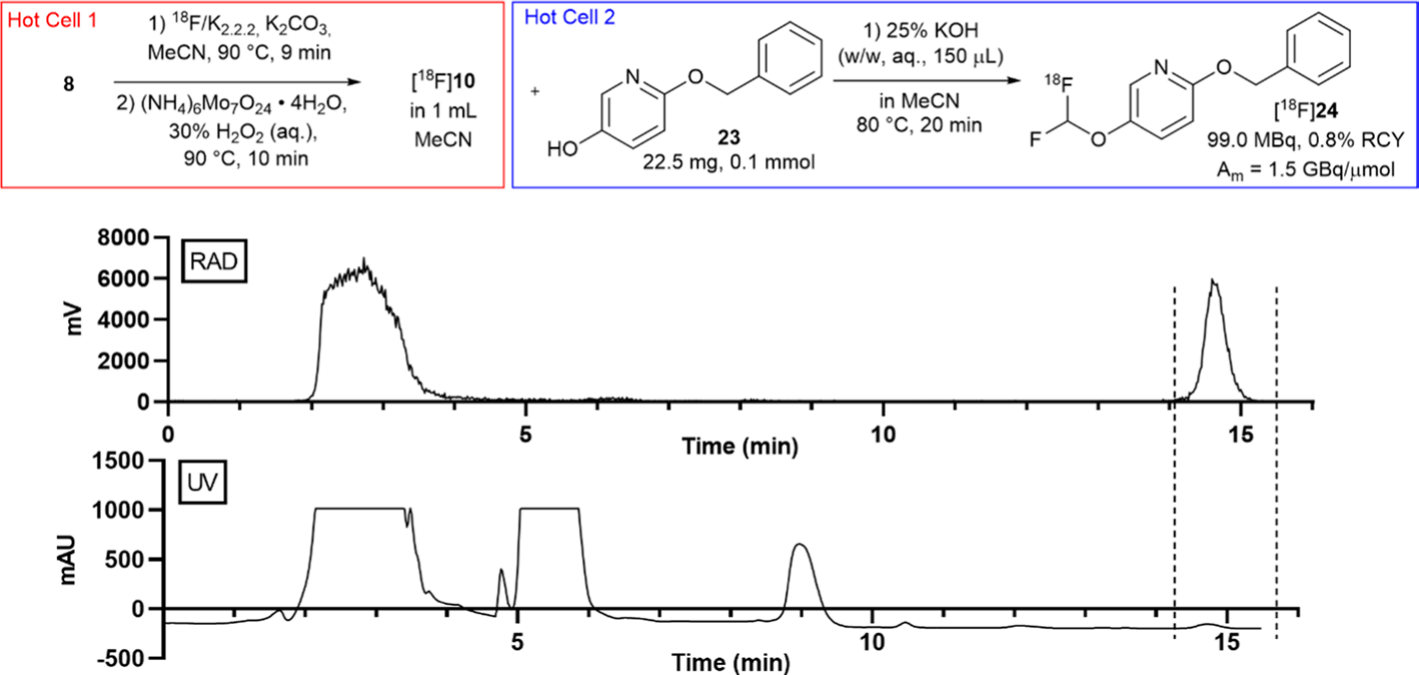
Fully automated radiosynthesis of [.^18F^]24 with semi-prep trace (dotted lines indicates collected product peak)

Using this set-up and starting from 22.8 GBq of [^18^F]fluoride, 99.0 MBq (0.8% RCY) of [^18^F]**24** could be radiosynthesised in greater that 99% RCP with a molar activity of 1.5 GBq/μmol and total synthesis time of 1 h and 43 min. (Fig. 7, Supplementary Figs. 12 and 13).

## Discussion

In this work we describe a simplified protocol for the radiosynthesis on [^18^F]difluorocarbene on a GE FX_FN_ module.

Initial attempts to obtain the requisite precursors** 3** and **8** from an α-fluorination of the relevant phenyl sulfide ethyl bromide with Selectfluor were unsuccessful, although such an approach had been successful on a phenyl sulfide ethyl chloride (Supplementary Fig. 1) (Kirihara et al. 2013). An alternative approach was devised based on a Hunsdiecker-type decarboxylative bromination developed by Tan and co-workers (Tan et al. 2017). Using Ag(Phen)_2_OTf as the catalyst and dibromoisocyanuric acid as brominating reagent, a range of primary, secondary and tertiary aliphatic (26 examples) acids successfully underwent decarboxylative bromination to give the desired alkyl bromides at room temperature, however none of the substrates tested contained a sulfur-containing moiety (Fig. 2). Nevertheless, this approach proved successful in the syntheses of precursors **3** and **8**.

When Tan and co-workers’ conditions were applied to acid **14**, no bromination was observed at ambient temperature. Instead, brominated fluoroacid was observed as confirmed by LC/MS analysis. Heating to 70 °C was required to trigger decarboxylation and conversion to the desired bromofluorosulfide precursor **4** (Supplementary Fig. 2). Contrastingly, 4-(chloro)phenyl acid **15** was highly reactive towards bromination under conditions reported by Tan and coworkers and consumed rapidly at room temperature resulting in extensive dibromination. Bromination was performed at 0 °C to limit polybromination with reaction times varying from 30 min to 2 h to afford **8**. The alternative synthesis pathway described herein to precursors **3** and **8** will hopefully encourage wider-spread adoption of [^18^F]difluorocarbene and [^18^F]difluoromethyl radical chemistry, given the lack of availability of dibromofluoromethane which was formerly required for synthesis of **3** and **8**. Notably, the yields obtained for the decarboxylative bromination approach described herein were comparable to those employing traditional S_N_2 substitutions with dibromofluoromethane in their syntheses of precursors **3** (16%) and **8** (32%), respectively (Sap et al. 2022; Trump et al. 2019).

With precursors **3** and **8** in hand, the radiosynthesis of [^18^F]**10** was first attempted on the GE FX_FN_ module, using reported conditions for the Trasis AllInOne cassette-based radiosynthesiser (Sap et al. 2022). The reported protocol calls for 0.02 mmol of the bromofluorosulfide (12 mg) to be reacted with azeotropically dried [^18^F]fluoride with K_2.2.2_ and potassium carbonate. Following a Halex reaction (Furuya et al. 2010) at 80 °C for 3 min in 1 mL of MeCN, a mixture of NaIO_4_ (52 mg) and RuCl_3_ · xH_2_O (2 mg) were added to the reactor and the oxidation is carried out at 40 °C for 5–10 min before dilution and semi-prep purification.

In our hands it was found that after the RuCl_3_/periodate oxidation, the diluted reaction mixture contained insoluble particulates which could not be dissolved in either aqueous or organic solvent (Fig. 4A). (Sap et al. 2022; Trump et al. 2019). It has been reported that the RuCl_3_ · xH_2_O/periodate oxidant system is unstable unless solvated with carbon tetrachloride and can form ruthenium carboxylate species (Carlsen et al. 1981). Furthermore, this precipitate routinely blocked the reactor needle and valves between the reactor and the intermediate vial (V12, Fig. 7) before the HPLC sample loop. Attempts to filter out the precipitate with various SPE cartridges and filters on transfer from the reactor to the intermediate vial proved unsuccessful.

Using the sulfide **8**, mCPBA, Oxone and (NH_6_)_4_Mo_7_O_24_ · 4H_2_O/H_2_O_2_ were screened to identify an alternative oxidant matrix which could achieve full conversion to the requisite sulfone in under 10 min. Only the molybdenum catalyst (NH_6_)_4_Mo_7_O_24_ · 4H_2_O—peroxide oxidant system used in the synthesis of Julia-Kocienski reagents (Sakaine et al. 2023) could furnish **10** at elevated temperatures (80–100 °C) (Supplementary Table 1). It should be noted that the oxidation of **8–10** with (NH_6_)_4_Mo_7_O_24_ · 4H_2_O/H_2_O_2_ in the presence of DMF as co-solvent was highly exothermic, resulting in vigorous spitting and should therefore not be used.

Prior to efforts in automating the radiosynthesis of [^18^F]**10** on the GE FX_FN_ module, attempts were made to optimise the conversion of **8** to [^18^F]**20** in a non-automated manner. Azeotropically dried fluoride ([^18^F]K_2.2.2_/K_2_CO_3_) was resuspended in MeCN and partitioned between V-vials to run parallel reactions under varied conditions. It was found that radiochemical conversions of **8** to [^18^F]**20** were lower in these parallel non-automated reactions when using literature conditions (6–12 mg **8**, 80 °C, MeCN, RCY^HPLC^ < 3%), suggesting that the amount of potassium carbonate may be important for reaction efficiency. Interestingly, use of an alternative TBA-OTs [^18^F]fluoride elution system did not furnish any of the desired product [^18^F]**20** (Orlovskaya et al. 2020).

In the development of the radiosynthesis of [^18^F]**4** and [^18^F]**10** described above it was found that the crude reaction mixture sampled prior to semi-prep purification contained [^18^F]fluoride and the product [^18^]fluorinated sulfones as the only radiochemicals. We reasoned it was possible to remove the semi-prep purification step and at this juncture without affecting subsequent ^18^F-difluoromethylation efficacy. [^18^F]**10** was chosen for further development owing to a parallel interest in our lab to develop a target radiotracer via difluorocarbene insertion. Where previously this has only been automated for cassette-based modules (namely, the GE FASTLab and Trasis AllinOne), this work demonstrates the feasibility of radiosynthesising [^18^F]difluorocarbene in a chromatography-free manner on non-cassette-based modules. Removal of the chromatography step shortened the overall synthesis time by around 25 min.

However, the high molar activity of 130 GBq/μmol obtained for [^18^F]**10** by Sap and co-workers could not be replicated, with A_m_ ranging from 1.5 to 11 GBq/μmol in this study. This may be due to several interacting factors, including differences in A_m_ of [^18^F]F^−^ used and the initial starting amounts of activity (Sap and co-workers used a starting activity of 135 GBq, compared with a maximum of 15.6 GBq in this work).

Next, we sought to validate that the [^18^F]**10** radiosynthesised from the chromatography-free synthesis described could undergo O–H difluorocarbene insertion on a radiosynthetically useful scale. The optimised literature conditions call for considerably large amounts of phenolic precursor (200 μmol, Fig. 7 (Sap et al. 2022) which greatly exceed those typically utilised in radiotracer development settings (5 to 100 μmol) (Webb et al. 2024). Beyond the involved and low-yielding radiosynthesis of [^18^F]**10** itself, the large amounts of phenolic precursor required for this ^18^F-difluoromethylation make wider implementation of this methodology challenging.

Results described herein suggest that it is possible to implement a method that is amenable to full automation in which the prescribed precursor amount is reduced by up to 40-fold whilst retaining useful radiochemical conversions (> 30% RCY^HPLC^). Indeed, the radiochemical conversion to two model compounds: [^18^F]**22** (selected from the original [^18^F]difluorocarbene insertion scope) (Sap et al. 2022), and compound [^18^F]**24** (a model compound for a radiosynthetic target in our lab) tolerated a reduction in required precursor amount.

Finally, it was desirable to fully-automate the [^18^F]difluorocarbene synthesis and insertion process to provide access to isolated ^18^F-difluoromethylated products without the need for manual manipulation of radioactivity. This was accomplished by using two neighbouring GE FX_FN_s. To the best of our knowledge, this represents the first fully-automated [^18^F]difluorocarbene insertion radiosynthesis; the entire process was complete within a single fluorine-18 half-life with only a single semi-prep HPLC purification step required. This demonstrates that it is indeed possible to fully automate the [^18^F]difluorocarbene insertion process on a non-cassette based system to provide quantities (> 20 MBq) of isolated fluorine-18 product which could then be used for preclinical biological investigations including small-animal PET imaging. Whilst this setup may not be available in every lab, similar automation can be achieved using two-reactor modular systems such as the FX2N and Synthra RNPlus modules. The discrepancy observed between the non-automated RCY^HPLC^ of 70% for [^18^F]**24** and low overall yield of 0.8% RCY obtained for the automated synthesis of [^18^F]**24** is likely an artefact of translating a non-automated reaction using small reagent volumes to an automated module. A per-radiotracer reaction and automation optimisation is recommended as the ^18^F-difluoromethylation efficiency appears to be highly sensitive to base amounts, reaction volumes, and precursor amounts.

## Conclusion

In summary, precursors **3** and **8** were synthesised in 21% and 54% yield using a decarboxylative bromination approach which circumvented the need for use of the ozone-depleting dibromofluoromethane. Difluoromethyl radical reagent [^18^F]**4** and the difluorocarbene reagent [^18^F]**10** were successfully radiosynthesised on a GE FX_FN_ module in 4% and 2% RCY, respectively, representing the first radiosynthesis of these reagents on this type of non-cassette based module. Furthermore, [^18^F]**10** could be radiosynthesised in up to 14% RCY on a GE FX_FN_ module without the need for semi-prep HPLC purification. SPE cartridge-purified [^18^F]**10** could be used to difluoromethylate **23** with moderate radiochemical conversions whilst greatly reducing (up to 40-fold) the amount of phenolic precursor called for in literature conditions. Finally, the three-step process was fully automated on two tandem GE FX_FN_ modules to yield useable amounts (> 20 MBq) of [^18^F]**24** in under two hours, providing a path forward for routine automated synthesis of radiotracers via [^18^F]difluorocarbene insertion.

## Methods

### Chemistry

#### General experimental

All chemicals and solvents (including anhydrous solvents) were purchased from Sigma Aldrich, ThermoFischer, Alfa Aesar, Fluorochem or AK Scientific without further purification. Aqueous solutions of brine, sodium bicarbonate, ammonium chloride and sodium thiosulfate were prepared as saturated solutions at room temperature. Where water is used during a reaction or work-up this refers to water purified by Merck Milipore Elix and Synergy UV. Unless otherwise stated, all reactions were carried out under an atmosphere of nitrogen using anhydrous solvents. Flash column chromatography was performed using high purity silica gel, pore size 60 Å, 220–240 mesh particle size and 35 −75 µm particle size. Reactions were monitored by analytical thin layer chromatography (TLC) on Merck Kieselgel 60 GF254 plates and liquid chromatography mass-spectrometry (LCMS). Compounds were visualised under ultraviolet fluorescence (*λ* = 254 nm). LC–MS data was obtained on a Waters Acquity-H/Xevo TQD LC–MS instrument using a Acquity UPLC® BEH C18 1.7 µm, 2.1 × 50 mm column. 4-Minute Method: 5–95% MeCN with 0.01% formic acid/H_2_O with 0.1% formic acid, flow rate: 0.6 mL/min. Water (solvent A) and acetonitrile (solvent B) containing 0.1% and 0.01% formic acid by volume, respectively, were used as the mobile phases at a flow rate of 0.6 mL/min. Gradient [time (min), %B solvent]: [0, 5], [2, 95], [3, 95], [3.1, 5], [4, 5]. The ESI source was operated in positive mode with a capillary voltage of 3 kV and a cone voltage of 40 V. Nitrogen was used as the nebulizer and desolvation gas. Probe temperature was set to 600 °C.

All nuclear magnetic resonance (^1^H, ^13^C and ^19^F) spectra were recorded at 298 K on a Bruker Avance III 300 MHz spectrometer. Data analysis was performed using Topspin 4.3.2 software. Spectra were obtained from the specified deuterated solvents in 5 mm diameter tubes. Chemical shifts are reported relative to residual solvent in CDCl_3_ (δ_H_ 7.26 ppm, δ_C_ 77.2 ppm) or *d*_6_-DMSO (δ_H_ 2.50 ppm, δ_C_ 39.5 ppm). Fluorine-19 NMR were referenced internally to CFCl_3_ according to Bruker’s lock specifications. Chemical shift is measured in ppm and quoted to the nearest 0.01 ppm for ^1^H NMR and 0.1 ppm for ^13^C and ^19^F NMR. Coupling constants are given to the nearest 0.1 Hz. Multiplicity is reported as: s = singlet, d = doublet, t = triplet, dd = doublet of doublets, dt = doublet of triplets, m = multiplet, br = broad, abq = AB quartet. Pople notation is used to designate certain common 2nd order systems including AA’XX’ and AA’BB’ systems. High resolution mass spectra were measured by the Yusuf Hamied Department of Chemistry Mass Spectrometry Section at the University of Cambridge in positive-ion mode using either a TOF or ESI HRMS. Melting points were determined using a Gallenkamp melting point apparatus.

#### Synthesis and characterisation

**2-(Benzo[d]thiazol-2-ylthio)−2-fluoroacetic acid (14)** 
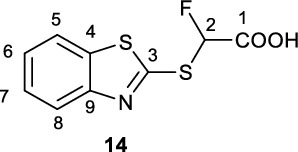


To a cooled (0 °C) slurry of 2-mercaptobenzothiazole (**1**, 5.38 g, 32.4 mmol) in ethanol (60 mL) was added NEt_3_ (4.5 mL, 3.3 g, 32.4 mmol, 1.0 eq.) which resulted in a clear yellow solution which was allowed to stir over an ice bath for 5 min under a flow of N_2_. To this solution was added ethylbromofluoroacetate (4.2 mL, 6.6 g, 35.9 mmol, 1.1 eq.) and the resultant reaction mixture was warmed to room temperature during which the consumption of the starting material was monitored *via* LC/MS. On reaction completion (18 h), water (30 mL) was added to the reaction mixture and acidified to pH 1 with 1 N HCl. The intermediate ester **12** was extracted into DCM (3 x 50 mL), and the organic extracts dried over Na_2_SO_4_. Solvent removal *in vacuo* yielded the intermediate ester **12** as a pale-yellow viscous oil which was used without further purification, assuming full conversion to the intermediate ester. The crude ester was dissolved in MeCN (80 mL), to which finely ground NaOH was added (4.12 g, 103 mmol, 3.2 eq.) and the reaction mixture allowed to stir at room temperature. After stirring for 18 h, ester **12** was fully consumed as determined by LC/MS ([M+H]+ m/z = 272.3, 2.07 min). Water (150 mL) was added to the reaction mixture and acidified to pH 0 with 1 N HCl. The product was extracted into DCM (3 x 50 mL) and dried over Na_2_SO_4_, filtered and solvent removed *in vacuo* to yield the title fluoroacetic acid **14** as a waxy brown solid which was triturated with pentane (5.33 g, 21.9 mmol, 68% yield over two steps).

^**1**^**H NMR** (300 MHz, *d*_6_-DMSO) δ ppm 8.05 (d, ^*3*^*J*_*HH*_ = 8.0 Hz, 1H, C5/C8-H), 7.97 (dd, ^*3*^*J*_*HH*_ = 8.0 Hz, 1H, C5/C8-H), 7.53 (dt, ^*3*^*J*_*HH*_ = 8.0 Hz, ^*4*^*J*_*HH*_ = 1.0 Hz, 1H, C6/C7-H), 7.47 (dt, ^*3*^*J*_*HH*_ = 8.0 Hz, ^*4*^*J*_*HH*_ = 1.0 Hz, 1H, C6/C7-H), 7.07 (d, ^2^*J*_*HF*_ = 50.3 Hz, 1H, C2-H).

^**13**^**C NMR** (75 MHz, *d*_6_-DMSO) δ ppm 166.5 (^*2*^*J*_*CF*_ = 26.0 Hz), 160.6 (d, ^*3*^*J*_*CF*_ = 3.2 Hz), 136.2, 127.2, 125.9, 122.6 (overlapping signals), 92.5 (^*1*^*J*_*CF*_ = 232 Hz)

^**19**^**F NMR** (282 MHz, *d*_6_-DMSO) δ ppm −159.9 (d, ^2^*J*_*HF*_ = −50.3 Hz, 1 F).

**LCMS** 1.37 min, *m/z* 244.2

**TLC** 50% EA/PE, R_f_ = 0.

**HRMS** [M + H] + = [C_9_H_7_FNO_2_S_2_] + requires, 243.9897, found 243.9909.

**2-((4-Chlorophenyl)thio)−2-fluoroacetic acid (15)**


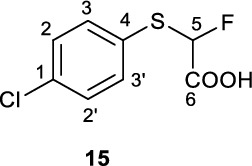
A round-bottom flask charged with 4-chlorothiophenol (**7**, 3.03 g, 21.0 mmol) and DMF (80 mL) was sparged with N_2_ for 30 min. The solution was then cooled on an ice-bath and NaH (60%, 1.05 g, 26.3 mmol, 1.2 eq.) was quickly added under N_2_ flow. The resultant suspension was stirred over ice for an additional 30 min until bubbling had subsided. To this slurry was added 2-ethylbromofluroacetate (2.7 mL, 4.3 g, 23.2 mmol, 1.1 eq) and the resultant reaction mixture was allowed to stir for 30 min (N.B. longer reaction times do not appear to be effective at pushing the reaction to further completion). The reaction was quenched by addition of sat’d NaHCO_3_ (150 mL) and the crude product (LC/MS: 2.16 min, no m/z observed) extracted into DCM (2 x 50 mL). Residual DMF was removed by washing the organic phase with aqueous LiCl (5% w/w, 3 x 50 mL), leaving the crude product **13** as a clear oil. The crude ester **13** was dissolved in MeCN (30 mL) to which was added NaOH (1.10 g, 0.0258 mmol, pellets ground to fine powder with mortar and pestle) and the resultant mixture allowed to stir under N_2_ at room temperature overnight, turning cloudier as the reaction progressed, until it was a thick slurry. The reaction was quenched by addition of saturated NaHCO_3_ (200 mL) and the pH adjusted to pH 7 with 4 N HCl. Unreacted 4-chlorothiophenol (LC/MS 2.06 min, no m/z observed) and other non-polar impurities was extracted into DCM (3 x 100 mL) and discarded. The aqueous phase was further adjusted to pH 0 with 4 N HCL and the product was extracted into DCM (3 x 100 mL), dried over Na_2_SO_4_ and solvent removed *in vacuo* to give acid **15** as a white crystalline solid (1.56 g, 7.1 mmol, 34%).

^**1**^**H NMR** (300 MHz, *d*_6_-DMSO) δ ppm 13.9 (brs, 1H, COOH), 7.51 (m, 4H, C2/2’/3/3’-H), 6.59 (d, 1H, ^2^*J*_*HF*_ = 50.8 Hz, C5-H).

^**13**^**C NMR** (75 MHz, *d*_6_-DMSO) δ ppm 168.0 (d, ^*2*^*J*_*CF*_ = 28.0 Hz), 135.0 (d, ^*4*^*J*_*CF*_ = 1.5 Hz), 134.5, 129.8, 129.4 (d, ^*3*^*J*_*CF*_ = 1.9 Hz), 93.0 (^*1*^*J*_*CF*_ = 228 Hz).

^**19**^**F NMR** (282 MHz, *d*_6_-DMSO) δ ppm −158.6 (d, *J*_*HF*_ = 50.8 Hz).

**MP** 84–86 °C.

**LCMS** 1.68 min, no m/z observable.

**TLC** 50% EA/PE, R_f_ = 0.

**HRMS** [M-H]^−^ = [C_8_H_5_ClFO_2_S]^−^ requires 218.9688, observed 218.9688.

**Silver(1 +), bis(1,10-phenanthroline-κN**^**1**^**,κN**.^**10**^**)-, (T-4)-, 1,1,1-trifluoromethanesulfonate (1:1) (17)**


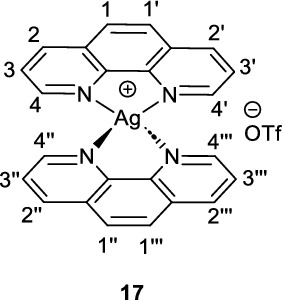
 [Ag(phen)_2_]OTf was synthesised using reported conditions (Ozawa and Kanai 2017). Briefly, a flask was charged with silver triflate (415.9 mg, 1.62 mmol, 1.0 eq.), 1,10-phenanthroline (**16**, 594.2 mg, 3.30 mmol, 2.0 eq.) and methanol (15 mL). The resultant slurry was stirred at room temperature for 3.5 hours after which the product was filtered off and washed with methanol three times and dried under vacuum to give the product **17** as a yellow solid (725.1 mg, 1.17 mmol, 73%). Spectroscopic data is in accordance with literature (Ozawa and Kanai 2017).

^**1**^**H **NMR (300 MHz, *d*_6_-DMSO) δ ppm 9.14 (dd, ^*4*^*J*_*HH*_ = 4.5 Hz, ^*5*^*J*_*HH*_ = 1.6 Hz, 4H), 8.76 (dd, ^*3*^*J*_*HH*_ = 8.2 Hz, ^*5*^*J*_*HH*_ = 1.6 Hz, 4H), 8.19 (s, 4H, C1-H, C1’-H, C1’’-H and C1’’’-H), 7.98 (dd, ^*3*^*J*_*HH*_ = 8.1Hz, ^*4*^*J*_*HH*_ = 4.5 Hz, 4H).

^**13**^**C NMR** (75 MHz, *d*_6_-DMSO) δ ppm 151.9, 142.4, 138.9, 129.5, 127.7, 125.4.

**LC/MS** 0.88 min, m/z [M-OTf]^+^ = 467.2

**HRMS** [M-OTf]^+^ = [C_24_H_16_AgN_4_]^+^ requires 467.0421, observed 467.044.

**2-((Bromofluoromethyl)thio)benzo[d]thiazole (3)**


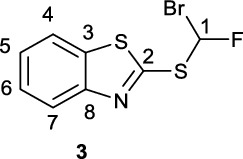
To a round bottom flask was added fluoroacetic acid **14** (332.1 mg, 1.36 mmol) and Ag(phen)_2_OTf (187.3 mg, 4 mol%). The flask was evacuated and refilled three times with N_2_ and subsequently charged with 1,2-dichloroethane (6 mL) under N_2_ flow and allowed to cool on an ice-bath for 5 min. To this solution was added dibromoisocyanuric acid (**18**, 183.7 mg, 0.65 mmol, 0.5 eq.) and the solution allowed to stir over an ice bath for 1 h before being allowed to warm to room temperature and was then further heated to 70 °C and stirred for 18 h. Over the course of the reaction, the reaction mixture yellowed and thickened to form a slurry. On full decarboxylation of the brominated intermediate observed *via* LC/MS (1.53 min, [M+H]+ m/z 322.1/324.1), water was added to the reaction mixture (30 mL) and product extracted into DCM (2 x 30 mL). The organic extracts were dried over sodium sulfate, filtered and dry-loaded onto SiO_2_ for FCC (10% EA/PE) to yield **3** as a yellow oil (81.1 mg, 0.29 mmol, 21%).

^**1**^**H NMR** (300 MHz, CDCl_3_) δ ppm 8.01 (d, ^*3*^*J*_*HH*_ = 8.0 Hz, 1H, C4/7-H), 7.96 (d, ^2^ *J*_*HF*_ = −55.2 Hz, 1H, C1-H), 7.84 (d, ^*3*^*J*_*HH*_ = 8.0 Hz, 1H, C4/7 -H), 7.51 (dt, ^*3*^*J*_*HH*_ = 8.0 Hz, ^*4*^*J*_*HH*_ = 1.2 Hz, 1H, C5/C6 – H), 7.42 (dt, ^*3*^*J*_*HH*_ = 8.0 Hz, ^*4*^*J*_*HH*_ = 1.2 Hz, 1H, C5/C6 – H).

^**13**^**C NMR** (75 MHz, CDCl_3_) δ ppm 160.1,152.8, 135.9, 126.7, 125.7, 122.9, 121.3, 89.2 (^*1*^*J*_*CF*_ = 297 Hz).

^**19**^**F NMR** (282 MHz, CDCl_3_) δ ppm −104.16 (d, *J*_*HF*_ = −55.2 Hz, 1 F).

**LC/MS** 2.17 min, [M + H] + m/z 278.0, 280.0

**TLC** 10% EA/PE, R_f_, 0.24.

**HRMS** [M + H] + = [C_8_H_6_BrNFS_2_] + requires 277.9106, found 277.9109.

**(Bromofluoromethyl)(4-chlorophenyl)sulfane (8)**


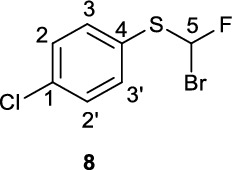
To a roundbottom flask was added fluoroacetic acid **15** (142.5 mg, 0.646 mmol) and Ag(phen)_2_OTf (**17**, 11.5 mg, 18.6 μmol, 3 mol%) and was evacuated and refilled three times with N_2._ 1,2-Dichloroethane (3 mL) was added under N_2_ flow and the resultant mixture cooled on an ice-bath. To this stirring slurry was slowly added dibromosocyanuric acid (**18**, 145.4 mg, 0.497 mmol, 0.8 eq.) and the resultant mixture was stirred at 0 °C for one hour. The reaction was monitored by LC/MS at 10-minute intervals as the starting material is highly reactive under these reaction conditions *N.B.* Leaving the reaction for prolonged periods (greater than 2 h) can result in the formation of various unwanted side products including those resulting from polybromination; therefore, it is imperative to the monitor reaction progress closely. Reaction times varied between 30 min and 2 h, reaction progress should be monitored closely *via* TLC or LC/MS when carrying out this reaction. When the reaction was deemed to have gone to completion, water (30 mL) was added to the reaction vessel and the product was extracted into DCM (3 x 20 mL). The organic extracts were washed with saturated sodium thiosulfate, dried over Na_2_ SO_4_, filtered and crude material loaded onto SiO_2_ for purification by FCC with 100% pentane. NB: a very large excess of SiO_2_ (>200:1 SiO_2_/product by weight) was used to enable separation from a less polar dibrominated side-product (R_f_ = 1.0 in 100% pentane). The final product **8** was isolated as a yellow oil (83.5 mg, 0.327 mmol, 51%). Spectroscopic data is in accordance with the literature (Sap et al. 2022).

^**1**^**H **NMR (300 MHz, CDCl_3_) δ ppm 7.52 (m[AA’BB’], 2H, C2/2’-H), 7.39 (m[AA’BB’], 2H, C(3,3’)-H), 7.22 (d, ^2^*J*_*HF*_ = 55.2 Hz, 1H, C5-H).

^**13**^**C NMR** (75 MHz, CDCl_3_) δ ppm 136.7, 136.0 (d,^*3*^*J*_*CF*_ = 1.6 Hz), 129.6, 128.7, 92.1 (^*1*^*J*_*CF*_ = 293 Hz)

^**19**^**F NMR** (282 MHz, CDCl_3_) δ ppm −100.00 (d, *J*_*HF*_ = 55.2 Hz).

**LCMS** 2.28 min, no m/z observable.

**TLC** 100% pentane, R_f_ = 0.7.

**HRMS** no *m/z* found, 3 methods tried.

**2-((Difluoromethyl)thio)benzo[d]thiazole (19)**


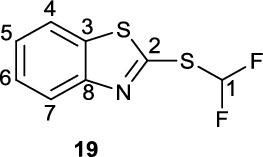
To a round-bottom flask was added 4-mercaptobenzothioazole (**1**, 346.9 mg, 2.074 mmol) which was dissolved in a 1:1 mixture of MeCN and 2.0 M KOH (8 mL) and the resultant mixture cooled on an ice-bath. To the cooled solution was added diethyl(bromodifluoromethyl)phosphonate (700 μL, 1.05 g, 3.94 mmol). The reaction mixture was stirred at 0 °C for 30 min after which the reaction mixture was warmed to room temperature and stirred for another 90 min until the reaction was determined to have gone to completion by LC/MS. The reaction was quenched by the addition of water (30 mL), the product extracted into DCM (3 x 10 mL) and the organic extracts dried over Na_2_SO_4_. The crude material was loaded onto SiO_2_ and purified by FCC (100% pentane to 10% EA/pentane) to give sulfide **19** as a thin yellow oil (317.0 mg, 1.459 mmol, 70%). Spectroscopic data in accordance with the literature (Luan et al. 2023).

^**1**^**H NMR** (300 MHz, CDCl_3_) δ ppm 8.01 (ddd, ^*2*^*J*_*HH*_ = 8.2 Hz, ^*3*^*J*_*HH*_ = 1.3 Hz, ^*4*^*J*_*HH*_ = 0.6 Hz,1H, C4-H or C7-H), Hz, 1.4 Hz, C4/7 -H), 7.84 (ddd, ^*2*^*J*_*HH*_ = 8.2 Hz, ^*3*^*J*_*HH*_ = 1.3 Hz, ^*4*^*J*_*HH*_ = 0.6 Hz, 1H, C4-H or C7-H), 7.74 (t, ^2^ *J*_*HF*_ = −55.2 Hz, 1H, C1-H), 7.50 (td, ^*3*^*J*_*HH*_ = 7.5 Hz, ^*4*^*J*_*HH*_ = 1.3 Hz, 1H, C5-H or C6 – H), 7.41 (td, ^*3*^*J*_*HH*_ = 8.0 Hz, ^*4*^*J*_*HH*_ = 1.2 Hz, 1H, C5-H or C6 – H).

^**19**^**F NMR** (282 MHz, CDCl_3_) δ ppm −93.2 (d, *J*_*HF*_ = 56.0 Hz, 2 F).

**LCMS** 2.07 min, m/z 218.0

**TLC** 100% pentane, R_f_ = 0.14.

**(Bromofluoromethyl)(4-chlorophenyl)sulfane (20)**


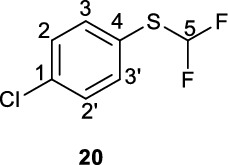
A solution of 4-chlorothiophenol (**7**, 1.10 g, 7.61 mmol) in a 1:1 mixture of MeCN and 2.0 M KOH (70 mL) was cooled on an ice-bath and sparged with N_2_ for 10 min. To this solution was added diethyl(bromodifluoromethyl)phosphonate (2.5 mL, 3.8 g, 14.1 mmol, 1.9 eq.) and the resultant reaction mixture stirred at 0 °C for 30 min. After 30 min the reaction was diluted with water (50 mL) and the product extracted into Et_2_O (3 x 50 mL). The organic extracts were washed with water (4 x 50 mL) and dried over Na_2_SO_4_. Solvent was removed to give sulfide **20** as a thin pale-yellow oil which was used without further purification (1.04 g, 5.34 mmol, 70%). Spectroscopic data is in accordance with the literature (Yang et al. 2017).

^**1**^**H NMR** (300 MHz, CDCl_3_) δ ppm 7.52 (m[AA’BB’], 2H, C2/C3-H), 7.37 (m[AA’BB’], 2H, C2/C3-H), 6.80 (t, ^*2*^*J*_*HF*_ = 56.7 Hz, 1H, C1-H).

^**13**^**C NMR** (75 MHz, CDCl_3_) δ ppm 136.8, 136.5, 129.6, 124.2, 120.3 (t, ^*1*^*J*_*CF*_ = 275 Hz).

^**19**^**F NMR** (282 MHz, CDCl_3_) δ ppm −91.69 (d, *J*_*HF*_ = −56.7 Hz, 2 F).

**LCMS** 2.13 min, no m/z observable.

**TLC** 100% PE, R_f_ = 0.48.

**HRMS** [M + H]^+^ = [C_7_H_6_ClF_2_S]^+^ requires 194.9842, found 194.9779.

**2-((Difluoromethyl)sulfonyl)benzo[d]thiazole (4)**


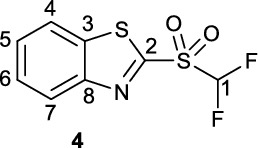
A flask was charged with sulfide **19** (0.317 g, 1.90 mmol) and dissolved in a 1:1:2 mixture of MeCN/CCl_4_/H_2_O). To this mixture was added RuCl_3_ · xH_2_O (9.0 mg, 0.043 mmol, 3 mol%) and NaIO_4_ (1.49 g, 6.967 mmol, 4.8 eq.) and the resultant reaction mixture allowed to stir at room temperature for 2 h. After complete oxidation was observed *via* LC/MS the reaction was quenched *via* the addition of water (15 mL) and the product was extracted into DCM (3 x 15 mL). The organic extracts were dried over Na_2_SO_4_ and solvent was removed *in vacuo* to give a clear oil which solidified on standing at 4 °C to give sulfone** 4** as a white crystalline solid (79.7 mg, 0.32 mmol, 17%). Spectroscopic data is in accordance with literature (Luan et al. 2023).

^**1**^**H NMR** (300 MHz, CDCl_3_) δ ppm 8.34 (m, 1H, C4-H or C7-H), 8.10 (m, 1H, C4-H or C7-H), 7.70 (m, 2H, C5-H and C6 – H), 6.58 (t, ^2^*J*_*HF*_ = −55.2 Hz, 1H, C1-H).

^**13**^**C NMR** (75 MHz, CDCl_3_) δ ppm 158.9, 153.0, 137.9, 129.0, 128.3, 126.3, 122.4, 114.5 (^*1*^*J*_*CF*_ = 288 Hz).

^**19**^**F NMR** (282 MHz, CDCl_3_) δ ppm −121.4 (d, *J*_*HF*_ = 53.1 Hz, 2 F).

**LCMS** 1.86 min, *m/z* 250.

**HRMS** [M + H] + = [C_8_H_8_F_2_NO_2_S_2_] + requires 249.9803, found 249.9800.

**TLC** 10% EA/PE, R_f_ = 0.08.

**1-Chloro-4-((difluoromethyl)sulfonyl)benzene (10)**


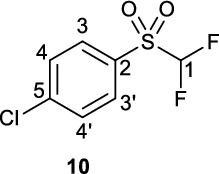
A round-bottom flask was charged with sulfide **20** (84.3 mg, 0.433 mmol) and reconstituted in MeCN (5 mL). To this solution as added aqueous H_2_O_2_ (3 mL, 30% w/w) and (NH_4_)_6_Mo_7_O_24_ · 4H_2_O (13.8 mg, 2.6 mol%) and the resultant reaction mixture stirred at 80 °C for 25 mins after which water was added (30 mL) and the product extracted into DCM (3 x 20 mL) and dried over Na_2_SO_4_. The solvent was removed *in vacuo* to give a yellow oil which solidified on standing at room temperature to give sulfone **10** as an off-white crystalline solid (41.8 mg, 0.18 mmol, 43%). Spectroscopic data conforms with literature (Sap et al. 2022).

^**1**^**H NMR** (300 MHz, CDCl_3_) δ ppm 7.93 (d [AA’BB’], ^*3*^*J*_*HH*_ = 8.8 Hz, 2H, C3-H/C3’-H), 7.64 (d (AA’BB’), ^*3*^*J*_*HH*_ = 8.8 Hz, 2H, C4-H/C4’-H), 6.19 (t, ^2^*J*_*HF*_ = −53.3 Hz, 1H, C1-H).

^**13**^**C NMR** (75 MHz, CDCl_3_) δ ppm (overlapping signals) 143.2, 132.1, 130.1, 114.6 (t, ^*1*^*J*_*CF*_ = 285 Hz).

^**19**^**F NMR** (282 MHz, CDCl_3_) δ ppm −121.2 (d, ^2^*J*_*HF*_ = −53.3 Hz, 2 F).

**LCMS** 1.85 min, no *m/z* observable.

**TLC** 10% EA/PE, R_f_ = 0.08.

**HRMS** [M + H] + = [C_7_H_6_ClF_2_O_2_S]^+^ requires 226.9740, found 226.9734.

#### X-ray crystallography

Crystals suitable for X-ray diffraction were grown by slow evaporation from CDCl_3_ at 4 °C. Single-crystal X-ray data were collected on a Bruker D8-QUEST diffractometer, equipped with an Incoatec IμS Cu microsource (λ = 1.5418 Å) and a PHOTON-III detector operating in shutterless mode. Structure and refinement details are included in the SI.

### Radiochemistry

#### General experimental

[^18^F]Fluoride was produced via the nuclear ^18^O(p,n)^18^F reaction in a target containing enriched [^18^O]H_2_O supplied from Secron or Rotex. The target was bombarded with a beam (20–60 μA) of protons accelerated to 16 MeV from a GE PETtrace cyclotron. At the end of irradiation, the radioactivity was transferred from the target to a V-vial using a flow of He (> 99.999% purity). Radioactivity was measured using a Capintec CRC-15R radioisotope dose calibrator.

Non-automated and automated radiolabelling reactions were performed in lead-shielded hotcells. Automated radiochemical reactions were performed on a TRACERlab FX_FN_ (GE Healthcare) automated module, operated by the accompanying software (TRACERlab FX_FN_). Semi-preparative HPLC was performed on the HPLC system associated with the GE TRACERlab FX_FN_. Analytical high-performance liquid-chromatography (HPLC) was performed on ThermoScientific Scientific Dioxex 3000 system coupled to a Bioscan standard radiation detector and data analysed using Chromeleon 7.2.

Decay-corrected radiochemical yields are reported as RCY (%), where RCY is defined as the activity of the isolated product (decay-corrected to the start of synthesis) divided by the starting activity of [^18^F]F^−^. Non-decay corrected yields are reported as RCY^ndc^ (%), where RCY^ndc^ is defined as the activity of the isolated product at the end of synthesis divided by the starting activity of [^18^F]F^−^. For non-automated radiochemical reactions where the final radiolabelled product is not isolated, reaction yield is given as RCY^HPLC^ (%), which is the peak area of the desired radiolabelled product divided by the sum of all peak areas in the HPLC radiochromatogram. Where appropriate, radiochemical yields and purities are reported as averages ± standard deviation.

#### Analytical HPLC methods

Method A: Column: Phenomenex SynergiTM 4 μM Hydro PR 80 Å 250 × 4.6 mm LC column. Flow rate = 1.0 mL per min. Solvent A: 25 mM ammonium formate (aq, pH 8); solvent B: MeCN. Gradient [time (min), %B solvent]: [0, 25], [1, 25], [10, 95], [14, 95], [17,25], [20. 25]. Injection volume: 20 μL.

Method B: Column: Luna 10 μM M C18(2) 100 Å 250 × 4.5 mm LC column. Flow rate = 1.5 mL per min. Solvent A: 25 mM ammonium formate (aq, pH 8); solvent B: MeCN. Gradient [time (min), %B solvent]: [0, 30], [3, 30], [7, 80], [10, 80], [11,30], [13, 80]. Injection volume: 20 μL.

#### Semi-preparative HPLC methods

Method A: Column: Phenomenex SynergiTM 4 um Hydro RP 80 Å 250 × 10 mm LC column. Flow rate = 4.0 mL per min. Eluent: 55% MeCN/25 mM ammonium formate (aq, pH 8). Isocratic conditions.

Method B: Column: Phenomenex SynergiTM 4 um Hydro RP 80 Å 250 × 10 mm LC column. Flow rate = 4.0 mL per min. Eluent: 65% MeCN/water. Isocratic conditions.

#### [^18^F]KF/K2.2.2 capture and drying

[^18^F]Fluoride was eluted form the cyclotron as an aqueous solution (2.0–2.5 mL) and was transferred to the GE TRACERlab FX_FN_ automated module with a flow of N_2_. The ^18^F in this solution was separated from the ^18^O-enriched water by passing through a pre-conditioned Sep-Pak Light (46 mg) Accel Plus QMA anion exchange cartridge (waters). The ^18^F fluoride as eluted into the reactor with a solution of Kryptofix 222 (7.5–8.5 mg in 850 μL MeCN) and K_2_CO_3_ (0.15 mL of a 10 mg/mL solution of water). [^18^F]fluoride was azeotropically dried at 100 °C under vacuum using three portions of anhydrous acetonitrile (3 × 333 μL) and then dried under He flow for 3–5 min at 120 °C to ensure complete drying.

#### Automated synthesis of [^18^F]4 and [^18^F]10 on the GE FXFN module with semi-prep purification

The automated synthesis of [^18^F]**4** and [^18^F]**10** was carried out on the GE TRACERlab FX_FN_ module. [^18^F]KF capture and drying was performed as outlined above. After drying, precursor **3** or **8** in MeCN (10–12 mg, 500 μL) was added to the reactor and heated to 90 °C for 9 min before cooling to 60 °C, the oxidant solution (40 mg (NH_4_)_6_Mo_7_O_24_ · 4H_2_O in 30% H_2_O_2_ (aq)) was then added to the reactor vessel and heated to 90 °C or 100 °C for (10 min). The reactor vessel was cooled to 50 °C before addition of 3 mL of the HPLC eluent (55% MeCN/25 mM ammonium formate). The contents of the reactor vessel were transferred to the intermediate vial and contents injected onto the HPLC (5 mL injection loop). The product peak was collected in diluted into water (50 mL) before passing through a Sep-Pak C18 Plus Light cartridge. The radiolabelled product was eluted with MeCN (1 mL) and dispensed into a neighbouring hot cell before QC analysis by radioHPLC (Method A, R_t_ (**4**) = 10.8 min, R_t_ (**10**) = 10.8 min). See SI for semi-prep traces.

#### Chromatography-free synthesis of [^18^F]10 on the GE FXFN module

The automated synthesis of [^18^F]**10** was carried out on the GE Tracerlab FX_FN_ module with a modified configuration such that only Vials 1 through to 6 were used for the radiosynthesis. The module was configured such that tube connecting the reactor vessel to the HPLC injection loop via the intermediate vial was elongated and connected to an aluminium cartridge in series with a SepPak plus C18 cartridge. These cartridges were loaded in the position where final product trapping and elution is usually performed from the HPLC collection flask. Instead, this configuration allows product to be trapped and eluted directly from the reaction vessel (refer to Supplementary Fig. 5 for a photo of modified FX_FN_ set-up.)

Once the module was reconfigured, the radiosynthesis of [^18^F]**10** was carried out as follows. [^18^F]KF capture and drying was performed as outlined above. After drying, precursor **8** in MeCN (500 μL) was added to the reactor and heated to 90 °C for 9 min before cooling to 60 °C. The oxidant solution was then added to the reactor vessel and heated to 90 °C for 20 min before the reactor vessel was cooled to 50 °C. Water from vial 5 (containing the maximum volume of *ca.* 15 mL of water) was added (5 mL) and the contents of the reactor vessel passed through the aluminium cartridge to remove unreacted fluoride and trapped on the SepPak C18 plus cartridge. Two washing steps were performed with the remaining water from vial 5 (2 × 5 mL) before elution of the product with MeCN (3 mL, vial 6) via the reactor vessel into the product vessel before being dispensed to a neighbouring hot cell for QC analysis by radioHPLC (HPLC Method A, R_t_ (rad) = 10.8 min).

#### Procedure for non-automated difluoromethylation of 21 with [.^18^F]10

[^18^F]**10** was radiosynthesised using the chromatography-free radiosynthesis described in Sect. “Chromatography-free synthesis of [18GF]10 on the GE FXFN module” formulated in MeCN (3 mL). Manual reactions were performed in an open lead-shielded hot cell. Reactions were performed in Wheaton V-vials (2 ml, sealed with a rubber septum) charged with **21** (23 μmol), MeCN (200 μL) and a mini stirrer bars. Aqueous base (2.5% KOH, w/w, 100 μL) was added immediately prior to addition of [^18^F]**10** (300 μL, 5–30 MBq) and stirred at 100 °C on the heating block under pressure (no vent needle, total reaction vol of 600 μL). After 20 min, the reaction vial was allowed to cool to room temperature for analysis by radioHPLC. Once cool, a small aliquot of the reaction mixture was quenched with water (to *ca.* 1:1 v/v) and analysed by radioHPLC for conversion to [^18^F]**22** (Method B, R_t_ = 13.3 min).

#### Procedure for non-automated difluoromethylation of 23 with [.^18^F]10

[^18^F]**10** was radiosynthesised using the chromatography-free radiosynthesis described in Sect. “Chromatography-free synthesis of [18GF]10 on the GE FXFN module” formulated in MeCN (3 mL). Manual reactions were performed in an open lead-shielded hot cell. Reactions were performed in Wheaton V-vials (2 ml, sealed with a rubber septum) charged with **23** (5–100 μmol) and a mini stirrer bar. Aqueous base (2.5% w/w KOH to 25% w/w KOH, 100 μL) was added immediately prior to addition of [^18^F]**10** (200 μL or 500 μL, 5–30 MBq), to give a total reaction volume of either 300 μL or 600 μL. The V-vials was stirred at 80 °C on the heating block for 10 min under pressure (no vent needle). After 10 min, the reaction vial was allowed to cool to room temperature for analysis by radioHPLC. Once cool, a small aliquot of the reaction mixture was quenched with water (to *ca.* 1:1 v/v) and analysed by radioHPLC for conversion to [^18^F]**22** (Method A, R_t_ = 12.4 min).

#### Procedure for fully-automated radiosynthesis of [.^18^F]24

Starting from 22.8 GBq of [^18^F]fluoride, [^18^F]**10** was radiosynthesised as outlined in Sect.“Chromatography-free synthesis of [18GF]10 on the GE FXFN module” (modified such that [^18^F]**10** was eluted with 1.5 mL of MeCN instead of 3.0 mL). [^18^F]**10** was dispensed into the reactor of a second neighbouring FX_FN_ module via Vygon tubing which was charged with **23** (22.5 mg, 0.11 mmol). To this mixture was added 25% KOH (w/w, 150 μL) from V3 and the sealed reactor vessel was stirred at 80 °C for 20 min. The reaction vessel was then cooled to 40 °C and diluted with semi-prep buffer (65% MecN/H2O) from V4. The contents of the reactor were transferred to the intermediate vial and purified by semi-prep column chromatography using Semi-Prep Method B. [^18^F]**24** was isolated as a peak eluting between 14 and 15 min and diluted into water (60 mL). The product was then trapped onto a pre-conditioned (5 mL EtOH then 10 mL water) SepPak light cartridge and eluted with MeCN (1 mL) from V9 into the product vial which was dispensed and analysed via radioHPLC (Method B, R_t_ = 10.8 min, yield = 99.0 MBq, RCY = 0.8%, RCP > 99%, molar activity = 1.5 GBq/μmol, total synthesis time = 1 h 43 min).

## Supplementary Information

Supplementary material 1

## Data Availability

Crystallographic data for **4**, **10** and **15** have been deposited at the Cambridge Crystallographic Data Centre (deposition numbers 2421950, 2,421,951, 2,421,952). The structures can be retrieved from: https://www.ccdc.cam.ac.uk/structures/. Supplementary Information is included in a separate pdf document.

## Abbreviations

A_m_: Molar activity
aq.: Aqueous
DBIA: Dibromoisocyanuric acid
DCE: 1,2-Dichloroethane
DCM: Dichloromethane
DMF: Dimethylformamide
DMSO: Dimethylsulfoxide
EA: Ethyl acetate
eq.: Equivalents
FCC: Flash column chromatography
HRMS: High resolution mass spectrometry
K_2.2.2_: Kryptofix 2.2.2
LC/MS: Liquid chromatography mass spectrometry
MeCN: Acetonitrile
ODS: Ozone depleting substance
PS: Petrol spirits
MA: Quaternary methyl ammonium
R_f_: Retention factor
R_t_: Retention time
RCP: Radiochemical purity
RCY: Decay-corrected radiochemical yield
RCY^HPLC^: HPLC radiochemical yield
RCY^ndc^: Non-decay corrected radiochemical yield
TLC: Thin layer chromatography
v/v: Volume/volume
w/w: Weight/weight

## Footnotes

1 †RCY = decay-corrected radiochemical yield (isolated product), RCY^ndc^ = non-decay corrected radiochemical yield (isolated product), RCY^HPLC^ = radiochemical yield determined from HPLC radiochromatogram (not isolated, crude).
