# Supplementary material for: Development of a halofluorocarbon, chromatography-free radiosynthesis of fluorine-18 difluorocarbene
Source: EJNMMI Radiopharm Chem. 2025 Jul 14;10:43. doi: 10.1186/s41181-025-00353-8 (PMC12260136; doi:10.1186/s41181-025-00353-8)
Supplement: Supplementary file 1 — Supplementary material 1 [file 41181_2025_353_MOESM1_ESM.docx]

**Supplementary Information**

**Development of a halofluorocarbon, chromatography-free radiosynthesis of fluorine-18 difluorocarbene**

C G F Dickmann,^1^ A D Bond,^2^ S Milicevic Sephton,^1^ F I Aigbirhio^1*^

^1^ Molecular Imaging Chemistry Laboratory, Wolfson Brain Imaging Centre, Department of Clinical Neurosciences, University of Cambridge, Cambridge Biomedical Campus, CB2 0SZ, U.K.

^2^ Yusuf Hamied Department of Chemistry, University of Cambridge, Lensfield Road, Cambridge, CB2 1 EW, U.K.

[*fia20@mesdchl.cam.ac.uk](mailto:*fia20@mesdchl.cam.ac.uk)

[Supplementary Figures and Tables 1](#_Toc191896997)

[Supplementary Synthesis Experimental 13](#_Toc191896998)

[NMR Data 14](#_Toc191896999)

[X-ray Crystallography Data 25](#_Toc191897000)

[Supplementary References 29](#_Toc191897001)

# Supplementary Figures and Tables

Supplementary Figure 1 Attempted synthesis of 8 via alpha-fluorination with Selectfluor per Kirihara et al.^1^


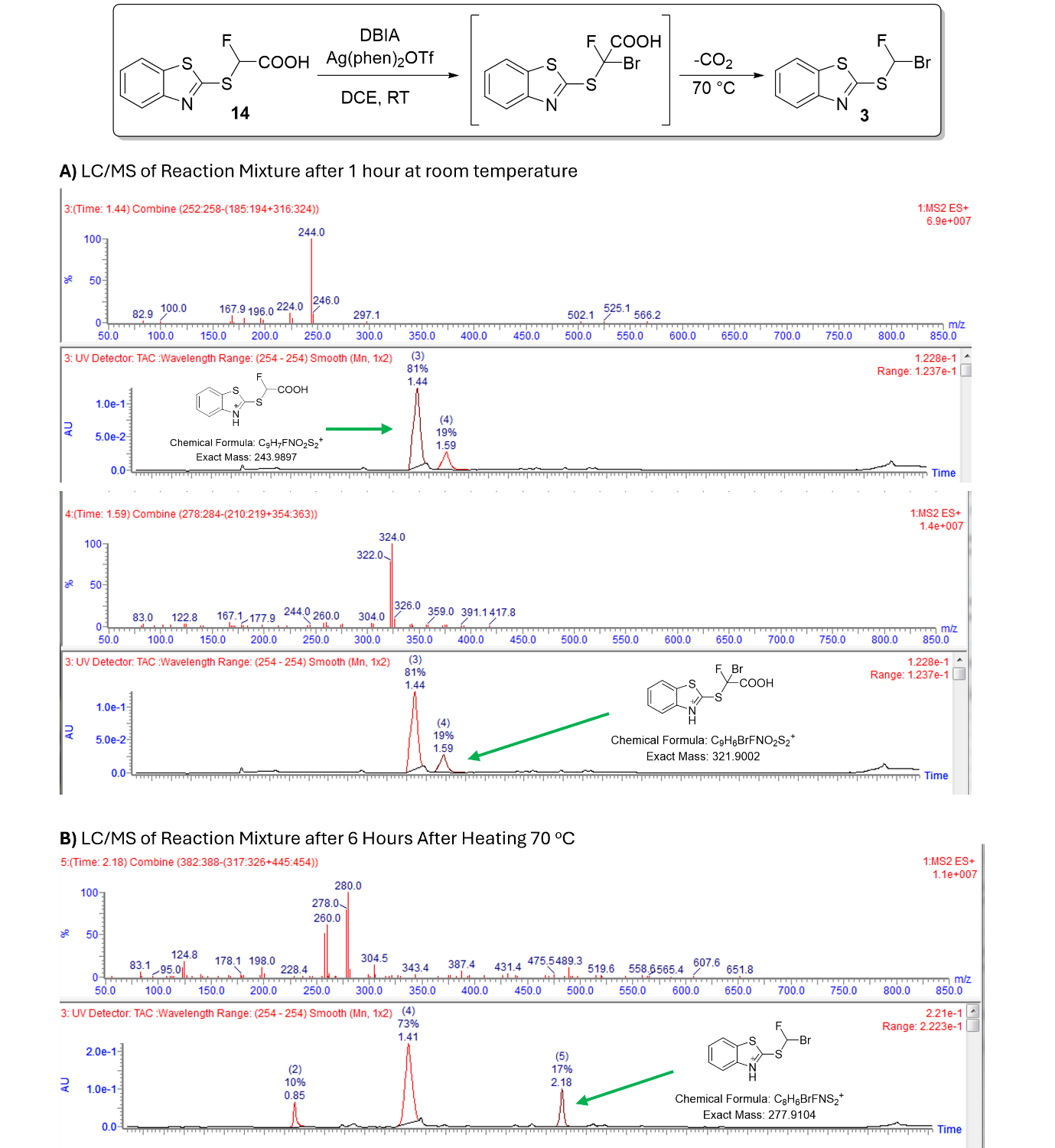


Supplementary Figure 2 LC/MS spectra of Hunsdiecker reaction of 14. (A) LC/MS of reaction mixture after 1 hour. Starting material and brominated intermediate observable. (B) LC/MS of reaction mixture after 6 hours after heating 70 °C. Decarboxylated product 3 observed.

Supplementary Table 1 Optimisation of oxidation conditions of 20 to 10. Reactions were carried out in the same Wheaton V-vials used for manual radiochemical reactions. Reaction progression was determined via LC/MS of the crude reaction mixture after 10 minutes.

| Entry | Mass of 20  (mg) | Vol of  30% H_2_O_2_ | Solvent | Volume of solvent | Total Rxn Volume | Catalyst loading | Temp (°C) | Time |
| --- | --- | --- | --- | --- | --- | --- | --- | --- |
| 1 | 12.3 | 0.1 mL | 5:2 MeCN/H_2_O | 1.4 mL | 1.5 mL | 17 mol% | 50 | **>10 min** |
| 2 | 12.7 | 0.5 mL | MeCN | 0.5 mL | 1.0 mL | 14 mol% | 80 | **>10 min** |
| 3 | 17.2 | 0.5 mL | MeCN | 0.5 mL | 1.0 mL | 16 mol% | 100 | **<10 min** |
| 4 | 15.5 | 0.5 mL | MeCN | 0.5 mL | 1.0 mL | 64 mol% | 80 | **<10 min** |
| 5 | 6.7 | 0.5 mL | MeCN | 1.0 mL | 1.5 mL | 67 mol% | 80 | **<10 min** |
| 6 | 3.2 | 1.0 mL | MeCN | 1.0 mL | 2.0 mL | 104 mol% | 80 | **>10 min** |
| 7 | 13.0 | 1.0 mL | DMSO | 1.0 mL | 2.0 mL | 76 mol% | 80 | **EXPLOSIVE** |
| 8 | 13.0 | 1.0 mL | DMF | 0.5 mL | 1.5 mL | 72 mol% | 50 | **>10 min** |
| 9 | 8.3 | 1.0 mL | DMF | 0.5 mL | 1.5 mL | 95 mol% | 60 | **>10 min** |
| 10 | 7.3 | 1.0 mL | DMF | 0.5 mL | 1.5 mL | 102 mol% | 70 | **<10 min** |
| 11 | 7.6 | 1.0 mL | DMF | 0.5 mL | 1.5 mL | 100 mol% | 80 | **< 5 min** |
| 12 | 11.8 | 1.0 mL | DMF | 0.5 mL | 1.5 mL | 74 mol% | 90 | **< 5 min** |

ssss


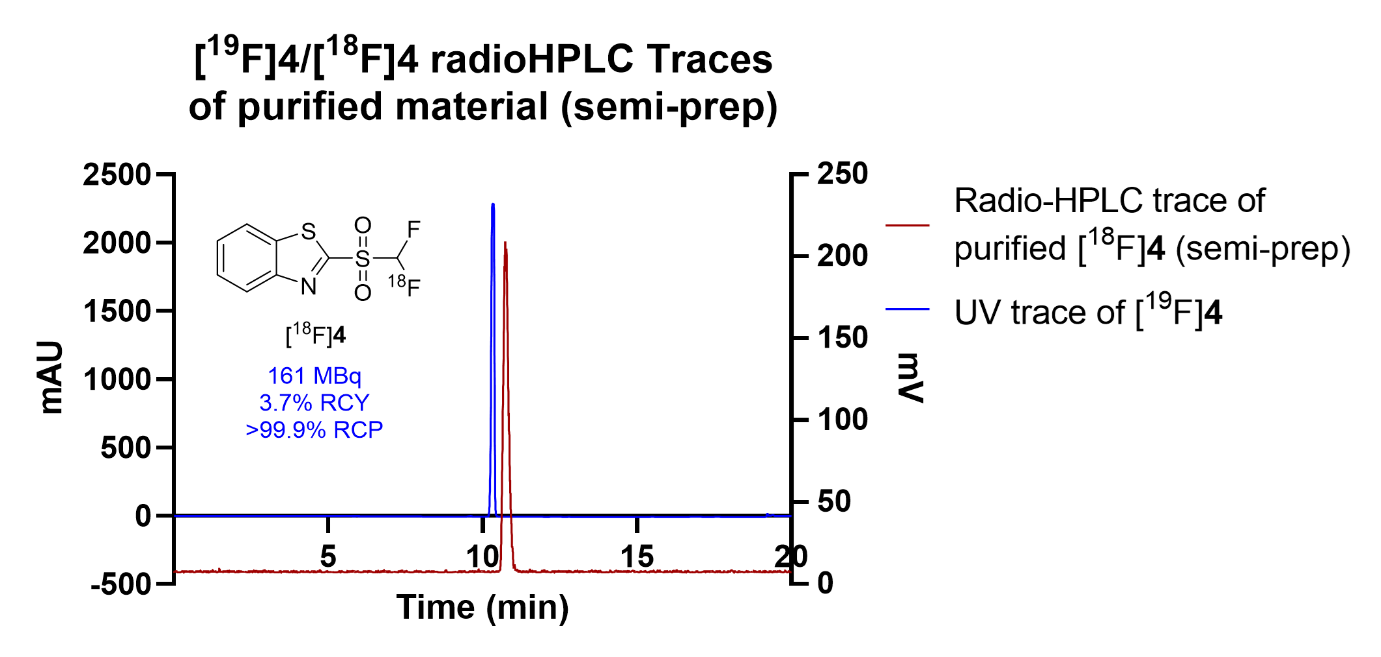


Supplementary Figure 3 RadHPLC trace (RAD and UV) of isolated [^18^F]4 material isolated from semiprep purification.


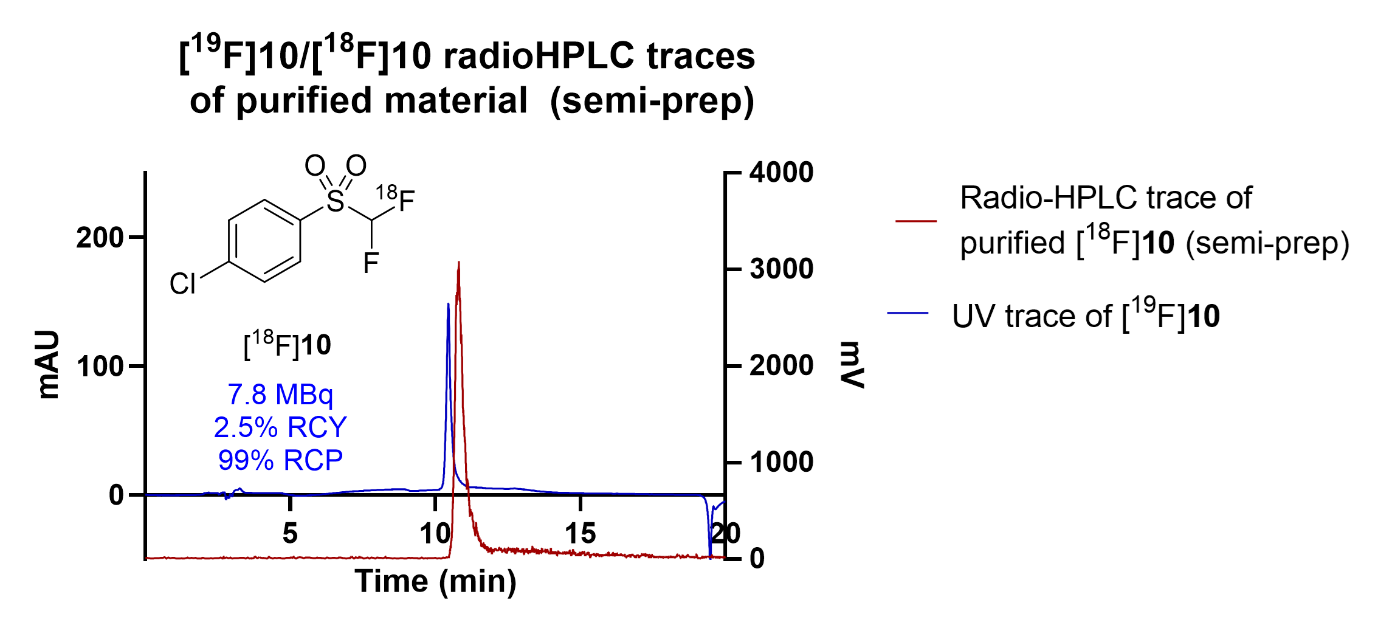


Supplementary Figure 4 RadHPLC trace (RAD and UV) of isolated [^18^F]10 material isolated from semiprep purification.


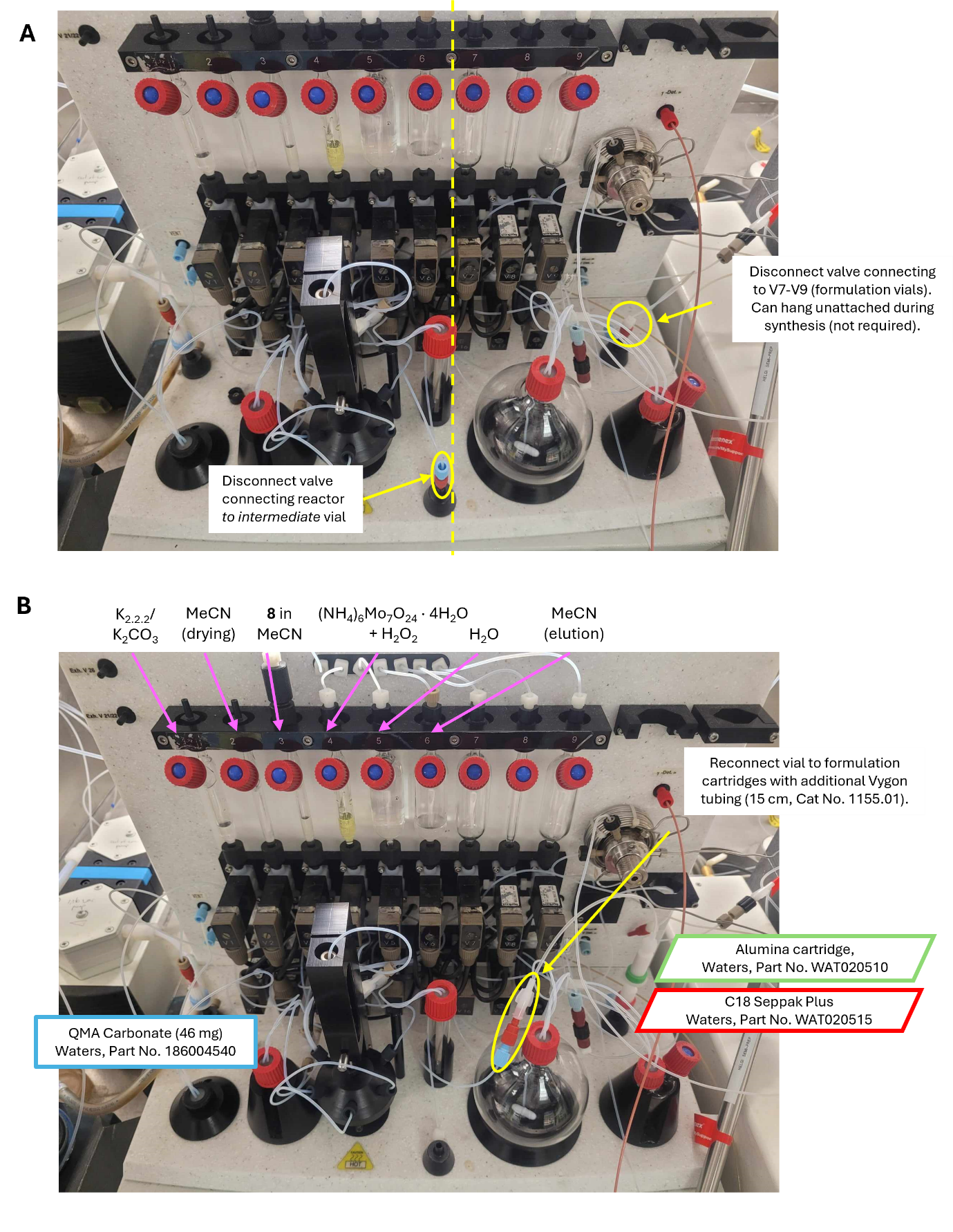


Supplementary Figure 5 Photo of FX_FN_ set-up for chromatography-free radiosynthesis of [^18^F]10 before (A) and after (B) modification from standard set-up.


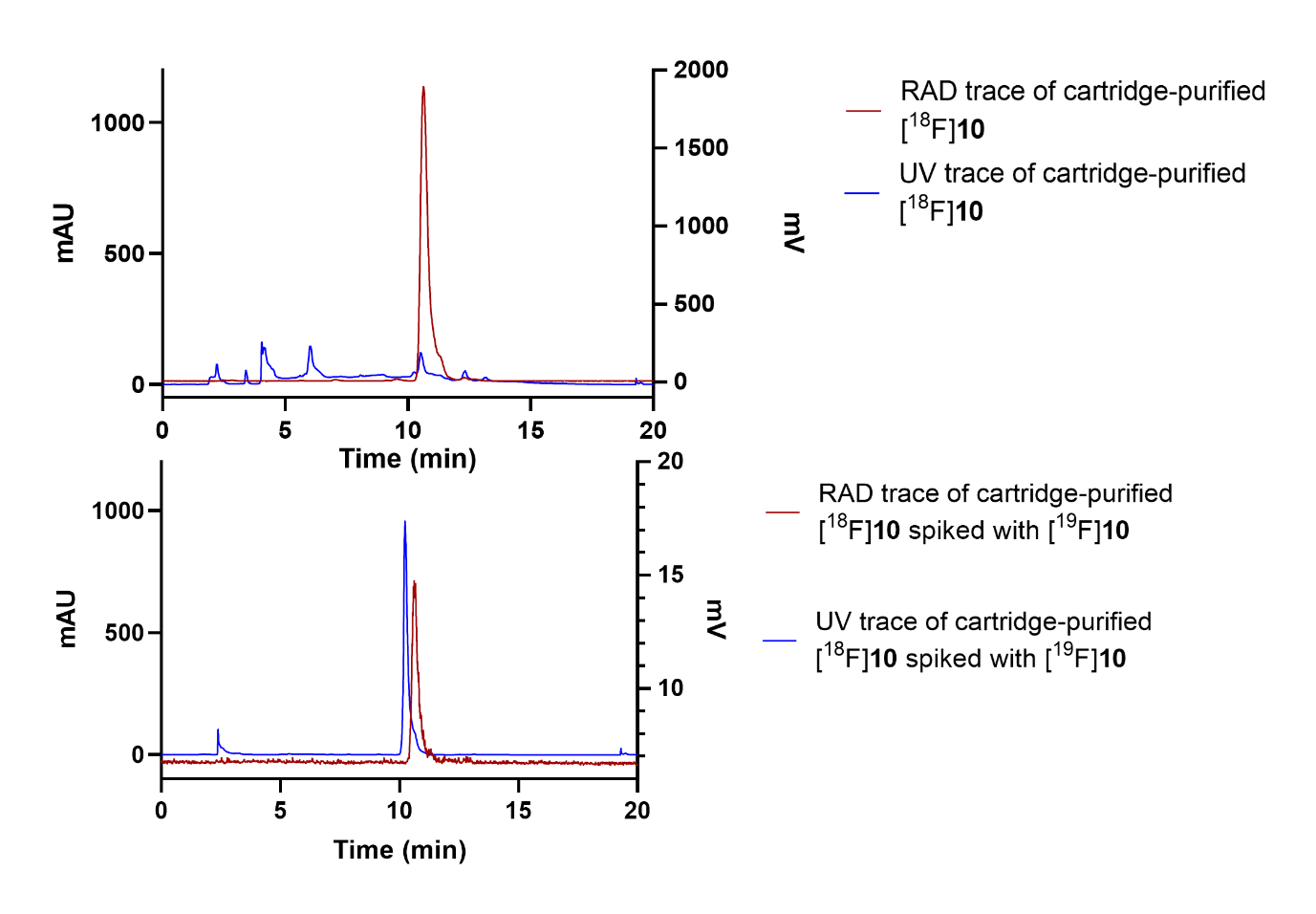


Supplementary Figure 6 RadHPLC QC trace of [^18^F]10 from chromatography-free radiosynthesis, neat (upper) and spiked with [^19^F]10 material for product idenfication (lower).


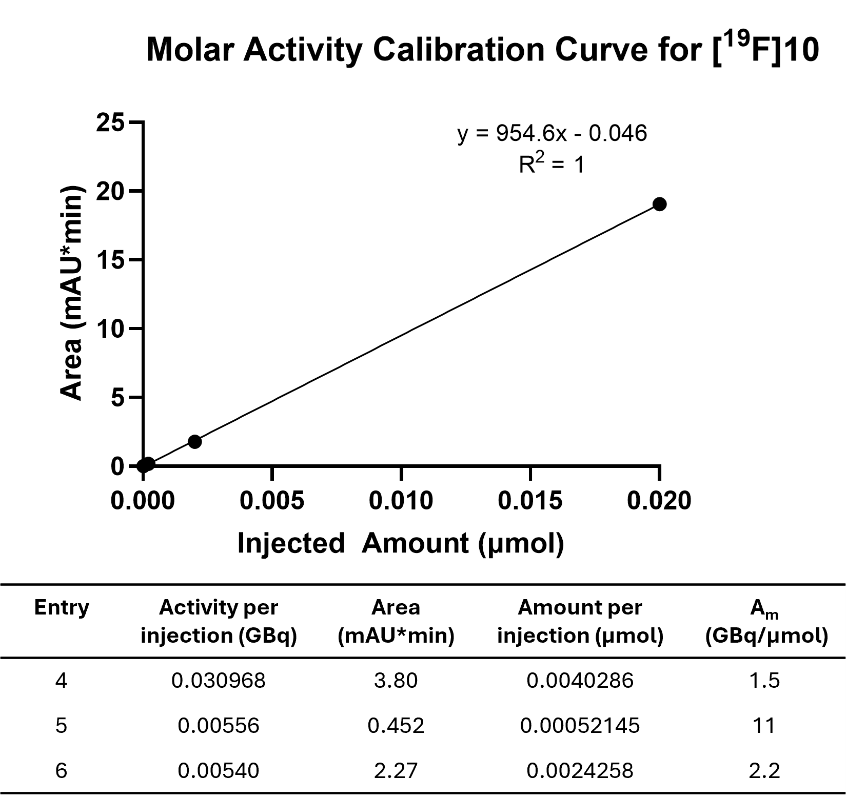


Supplementary Figure 7 Molar activity calibration curve of [^19^F]10 and A_m_ calculations for [^18^F]10.

| Entry | Amount Precursor 8 | Starting Activity | Time Halex | Time Oxidation | Yield | Total Synthesis Time | RCY^ndc^ | RCY | RCP | A_m_  (GBq/μmol) |
| --- | --- | --- | --- | --- | --- | --- | --- | --- | --- | --- |
| 1 | 8.0 mg | 5.4 GBq | 7 min | 12 min | 301 MBq | 45 min | 5.50% | 8.8% | 98% | n.d. |
| 2 | 9.8 mg | 6.70 GBq | 7 min | 10 min | 565 MBq | 42 min | 8.80% | 10.10% | 99% | n.d. |
| 3 | 9.8 mg | 12.7 GBq | 7 min | 11 min | 778 MBq | 1hr 24 min* | 7.9% | 10.40% | 93% | n.d. |
| 4 | 11.2 mg | 8.88 GBq | 9 min | 9 min | 894 MBq | 48 min | 10.10% | 14% | 98% | 1.5 |
| 5 | 10.2 mg | 13.3 GBq | 7 min | 10 min | 834 MBq | 49 min | 6.30% | 8.60% | 93% | 11 |
| 6 | 11.5 mg | 15.6 GBq | 9 min | 10 min | 810 MBq | 1h20min* | 5.2% | 8.6% | 98% | 2.2 |
|  |  |  |  |  |  | 46 ± 3 min | 7.3% ± 1.8% | 10.1% ± 1.9% | 97% ± 3% |  |

Supplementary Table 2 Summary table of results from chromatography-free radiosynthesis of [^18^F]10. *Excluded as product retrieval was delayed owing to safety lock on hot cell. n.d., not determined.


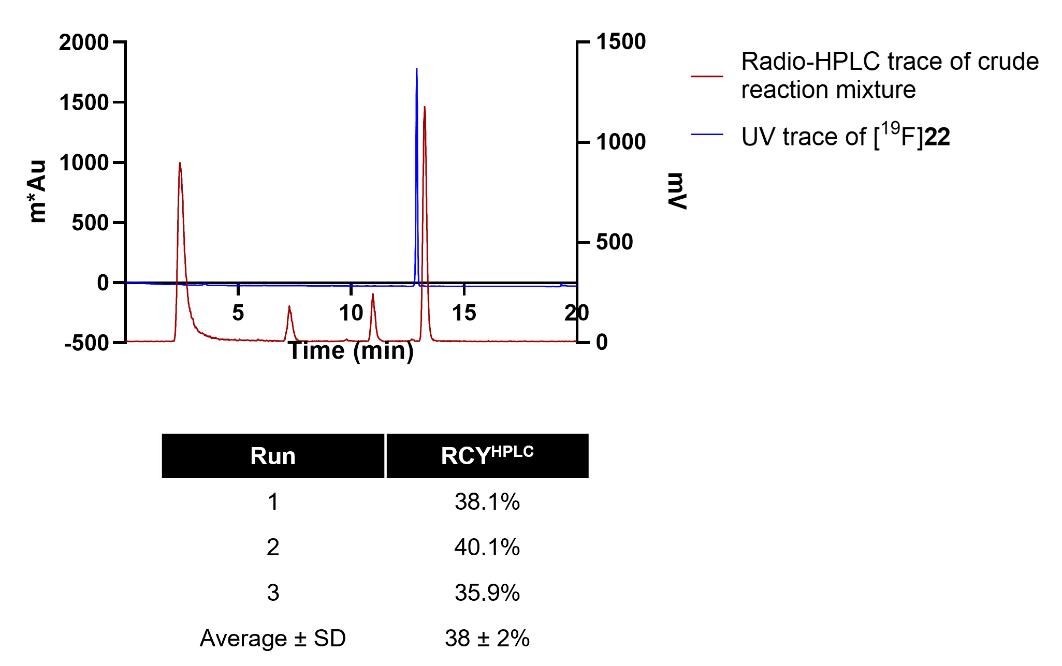


Supplementary Figure 8 RadHPLC trace (RAD and UV) for difluoromethylation of 21 to [^18^F]22 with [^18^F]10 depicted in Figure 6.


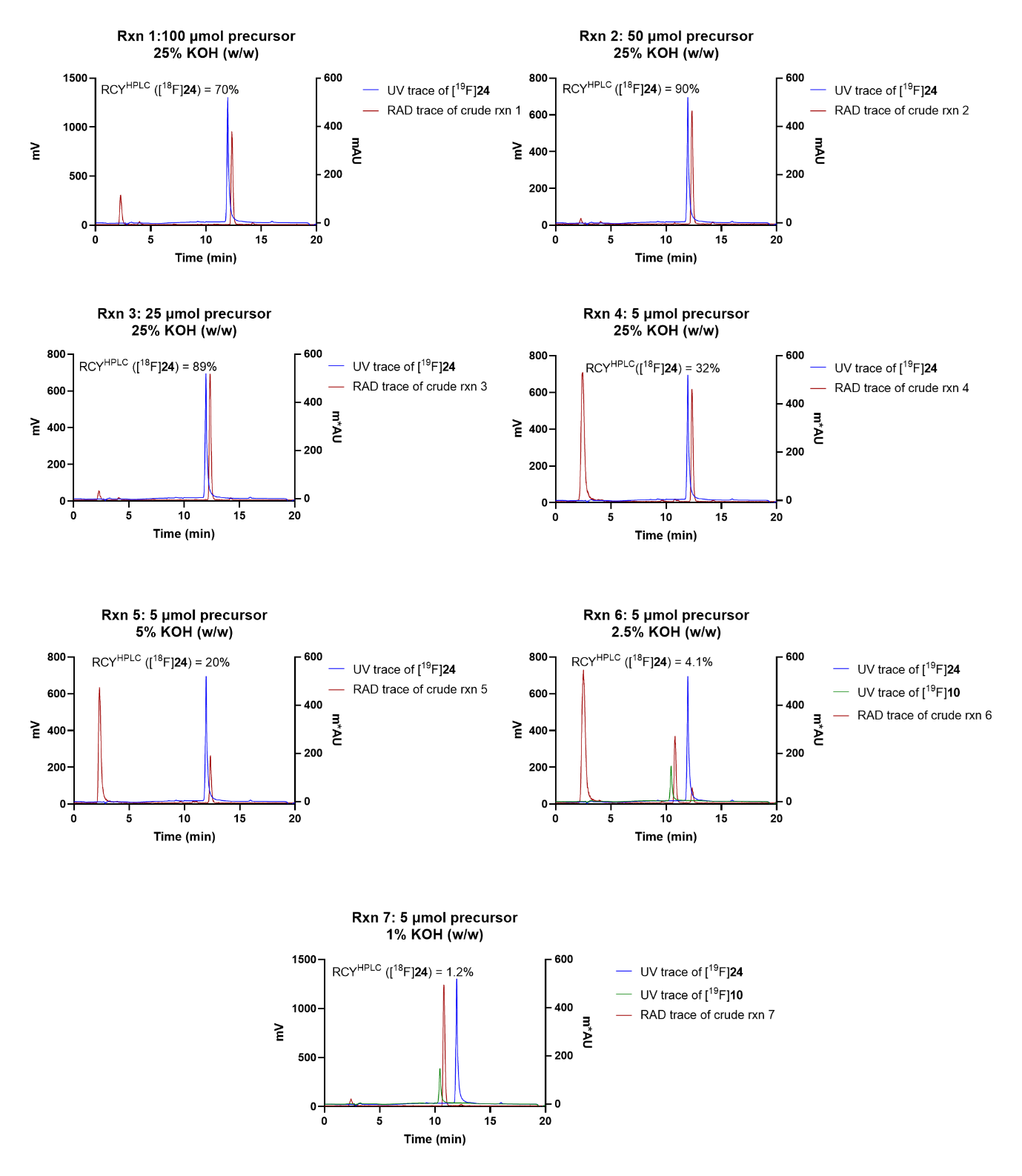


Supplementary Figure 9 RadHPLC trace (RAD and UV) for difluoromethylation of 23 to [^18^F]24 with [^18^F]10 depicted in Figure 6.

Supplementary Figure 10 Synthesis of non-labelled reference compounds 22 and 24.


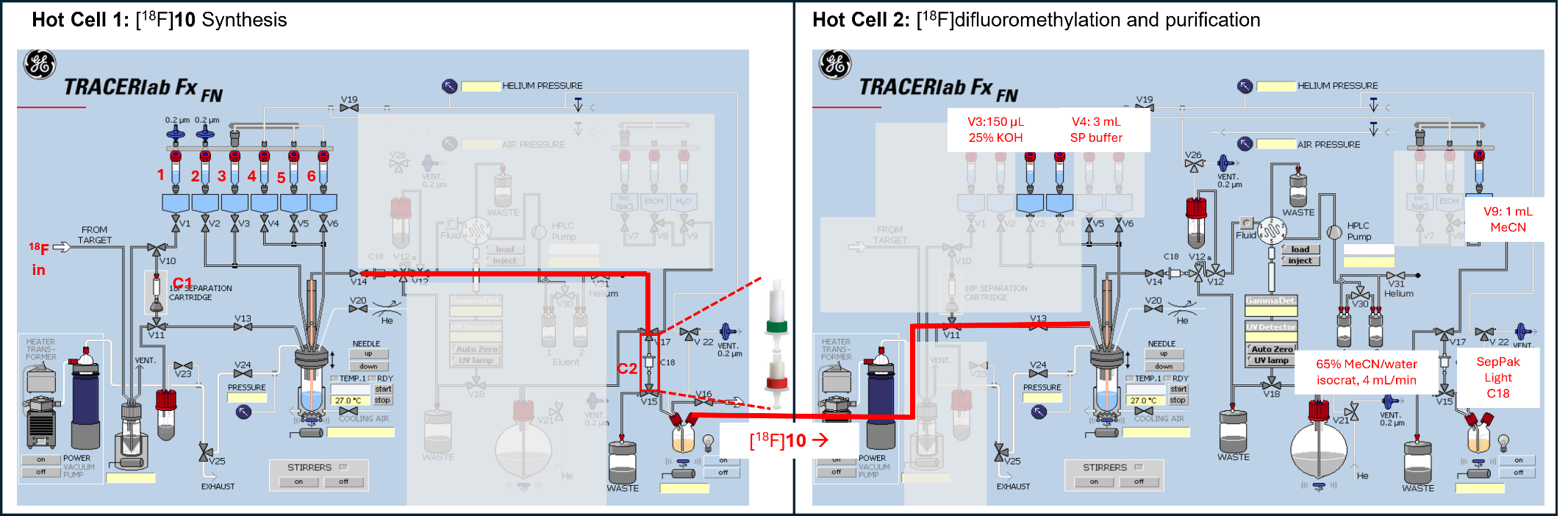


Supplementary Figure 11 Schematic of dual-FX_FN_ set-up for fully automated [^18^F]difluorocarbene synthesis, insertion to yield [^18^F]24 and purification.


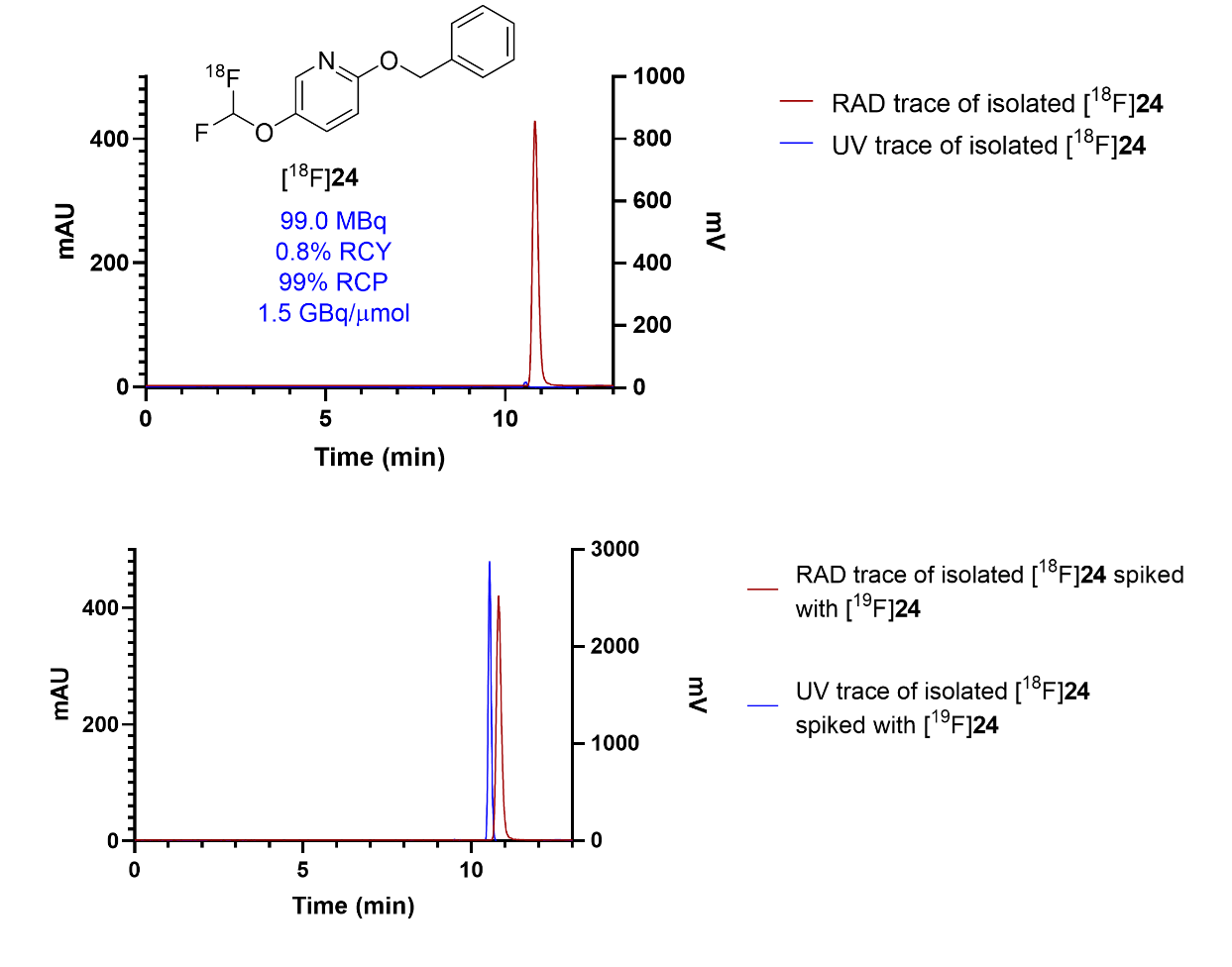


Supplementary Figure 12 radHPLC QC trace of [^18^F]24 isolated from fully automated (upper) and spiked with [^19^F]24 material for product idenfication (lower).


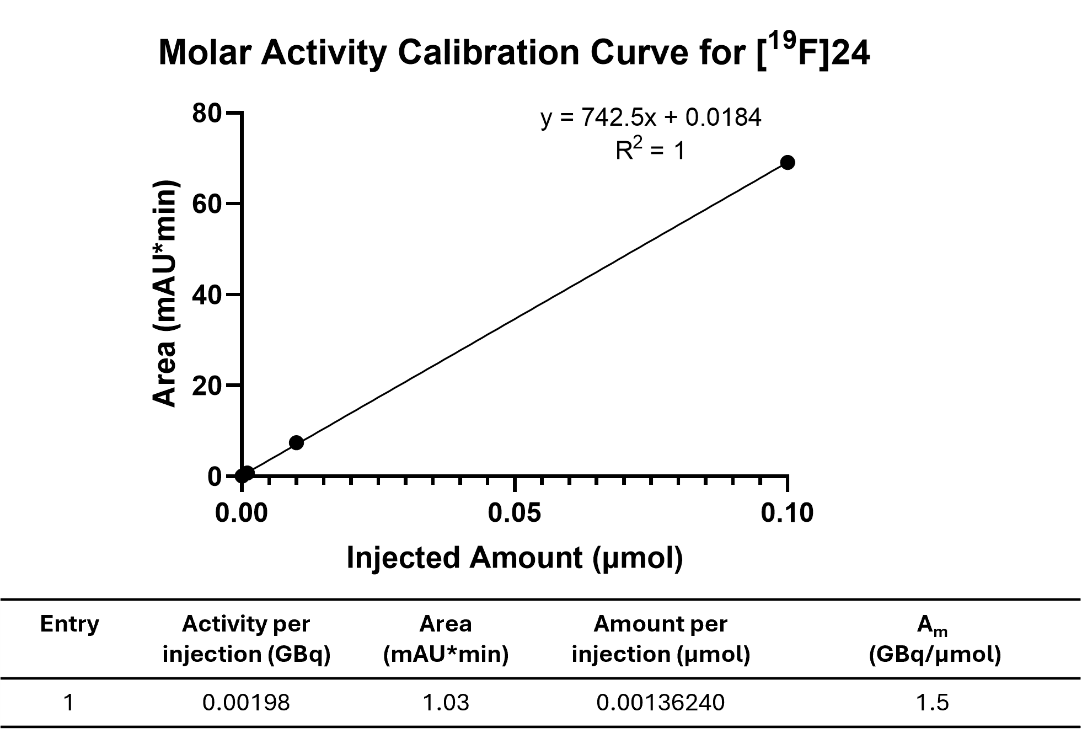


Supplementary Figure 13 Molar activity calibration curve for [^19^F]24 and A_m_ calculations for [^18^F]24.

# Supplementary Synthesis Experimental

**5,7-dichloro-8-(difluoromethoxy)quinoline (22)**

To a solution of chloroxine (**21**, 253.2 mg, 1.2 mmol, 1.0 eq.) in MeCN (5 mL) was added KOH (2 M, 5 mL) and the resultant mixture was stirred on an ice-bath for 5 minutes to allow for cooling. Diethyl(bromodifluoromethyl)phosphonate (1.503 g/mL, 600 μL, 901.8 mg, 3.4 mmol, 2.8 eq.) was added and the resultant reaction mixture was stirred for 30 minutes on an ice-bath before warming to room temperature. After two hours of stirring at room temperature, H_2_O (20 mL) was added to the reaction mixture and the product extracted into diethyl ether (2 x 30 mL), dried over Na_2_SO_4_ and loaded onto SiO_2_. Purification by FCC (100% pentane to 10% EA/pentane) gave **22** as a white crystalline solid (34.5 mg, 0.131 mmol, 11%). Spectroscopic data is in accordance with the literature.^2^

**^1^H NMR** (300 MHz, CDCl_3_) δ ppm 9.0 (dd, *^4^J_HH_* = 4.2 Hz, *^5^J_HH_* = 1.7 Hz, 1H, C2-H), 8.56 (dd, *^3^J_HH_* = 8.6 Hz, ^5^J_HH_ = 1.7 Hz, 1H, C3-H), 7.71 (s, 1H, C6-H), 7.59 (dd, *^3^J_HH_* = 8.6 Hz, *^4^J_HH_* = 4.2 Hz, 1H, C2-H), 7.34 (t, ^2^*J_HF_* = 76.7 Hz, 1H, C10-H).

**^13^C NMR** (75 MHz, CDCl_3_) δ ppm 151.6, 142.8 (t, *J* = 3.5 Hz), 142.3, 133.6 128.9, 128.1, 128.0, 126.2, 122.6, 116.9 (t, *^1^J_CF_* = 262 Hz).

**^19^F NMR** (282 MHz, CDCl_3_) δ ppm -82.4 (d, *^2^J_HF_* = 76.2 Hz*,* 2F)

**LCMS** 2.19 min, 264.9.

**TLC** 10% EA/PE, R_f_ = 0.27

**HRMS [**M+H]^+^ = [C_10_H_6_Cl_2_F_2_NO]+ requires 263.9789, found 263.9791.

**2-(benzyloxy)-5-(difluoromethoxy)pyridine (24)**

A flask was charged with 6-(benzyloxy)pyridin-3-ol (**23**, 196.9 mg, 0.979 mmol), sodium difluorochloroacetate (223.2 mg, 1.46 mmol, 1.5 eq.) and K_2_CO_3_ (207.3 mg, 1.50 mmol, 1.52 eq.) and constituted in DMF (4 mL). The resultant mixture was stirred at 80 °C for 1 hour after which the reaction mixture was cooled and acidified to pH 1 with 5 M HCl­_(aq). ­_The product was extracted into DCM (3 x 20 mL), dried over Na_2_SO_4_, filtered and loaded onto SiO_2_ for FCC (5% EA/pentane) to give the difluoromethylated product **24** as a clear oil (108.7 mg, 0.413 mmol, 42%).

**^1^H NMR** (300 MHz, CDCl_3_) δ ppm 8.05 (dd, *^4^J_HH_* = 3.0 Hz, *^5^J_HH_*  = 0.45 Hz, 1H, C9-H), 7.47 – 7.30 (m, 6H, C1-H, C2-H, C2’-H, C3-H, C3’H, C7-H), 6.80 (dd, *^3^J_HH_*  = 8.9 Hz, *^5^J_HH_* = 0.45 Hz, C6-H), 6.45 (t, 1H, ^2^*J_HF_* = 73.4 Hz).

**^13^C NMR** (75 MHz, CDCl_3_) δ ppm 161.2, 142.1 (t, *^3^J_CF_* = 262 Hz), 138.9, 137.0, 132.3, 128.5, 128.0, 128.0, 115.6 (t, *^1^J_CF_* = 262 Hz), 111.8, 68.0

**^19^F NMR** (282 MHz, CDCl_3_) δ ppm -81.1 (d, *J_HF_* = 73.4 Hz, 2F­).

**LCMS** 2.15 min, [M+H]+ = 252.1

**TLC** 10% EA/PE, R_f_ = 0.35

**HRMS** [M+H]+ = [C_13_H_12_F_2_NO_2_]+ requires 252.0831, found 252.0832.

# NMR Data


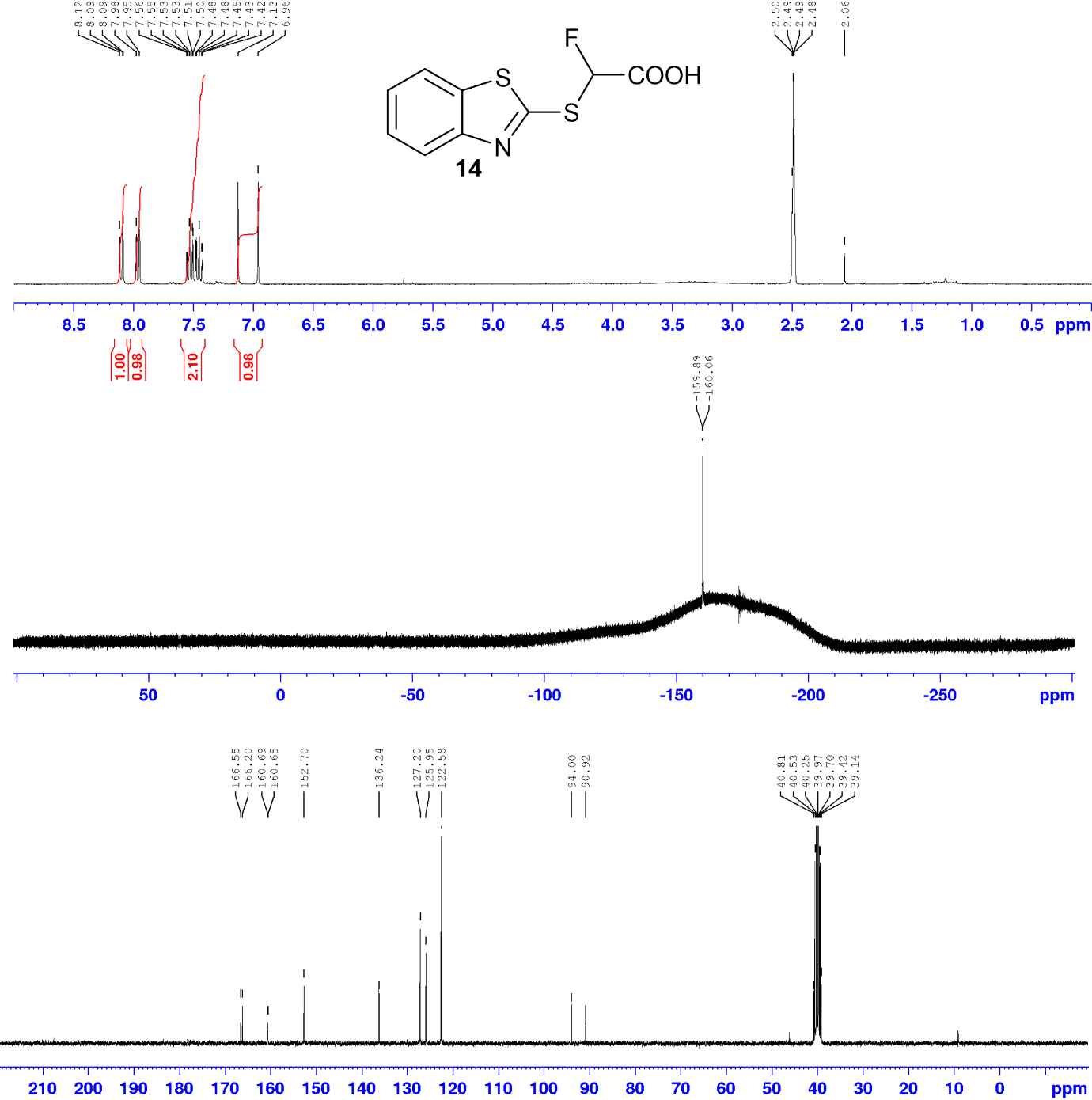


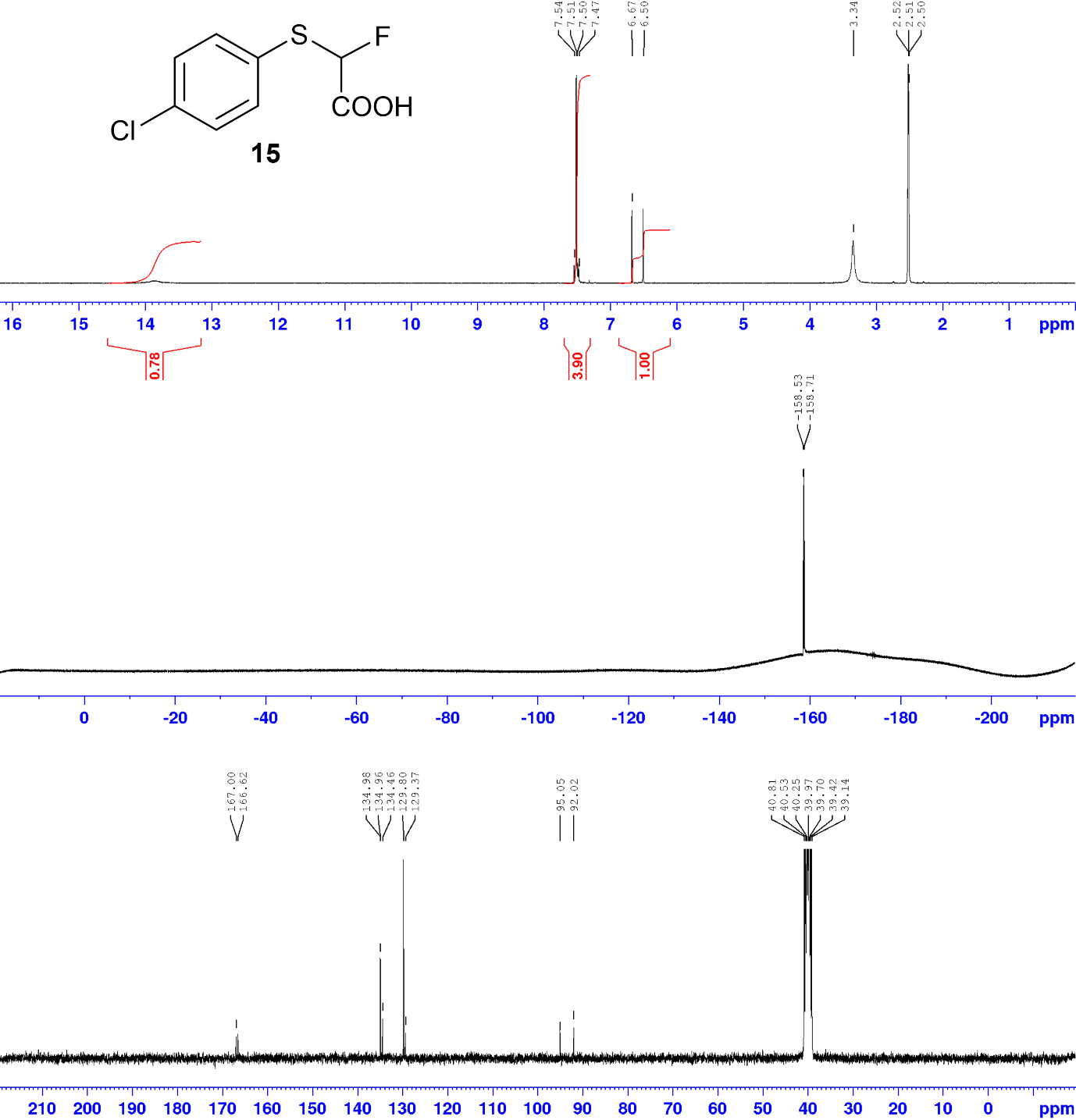


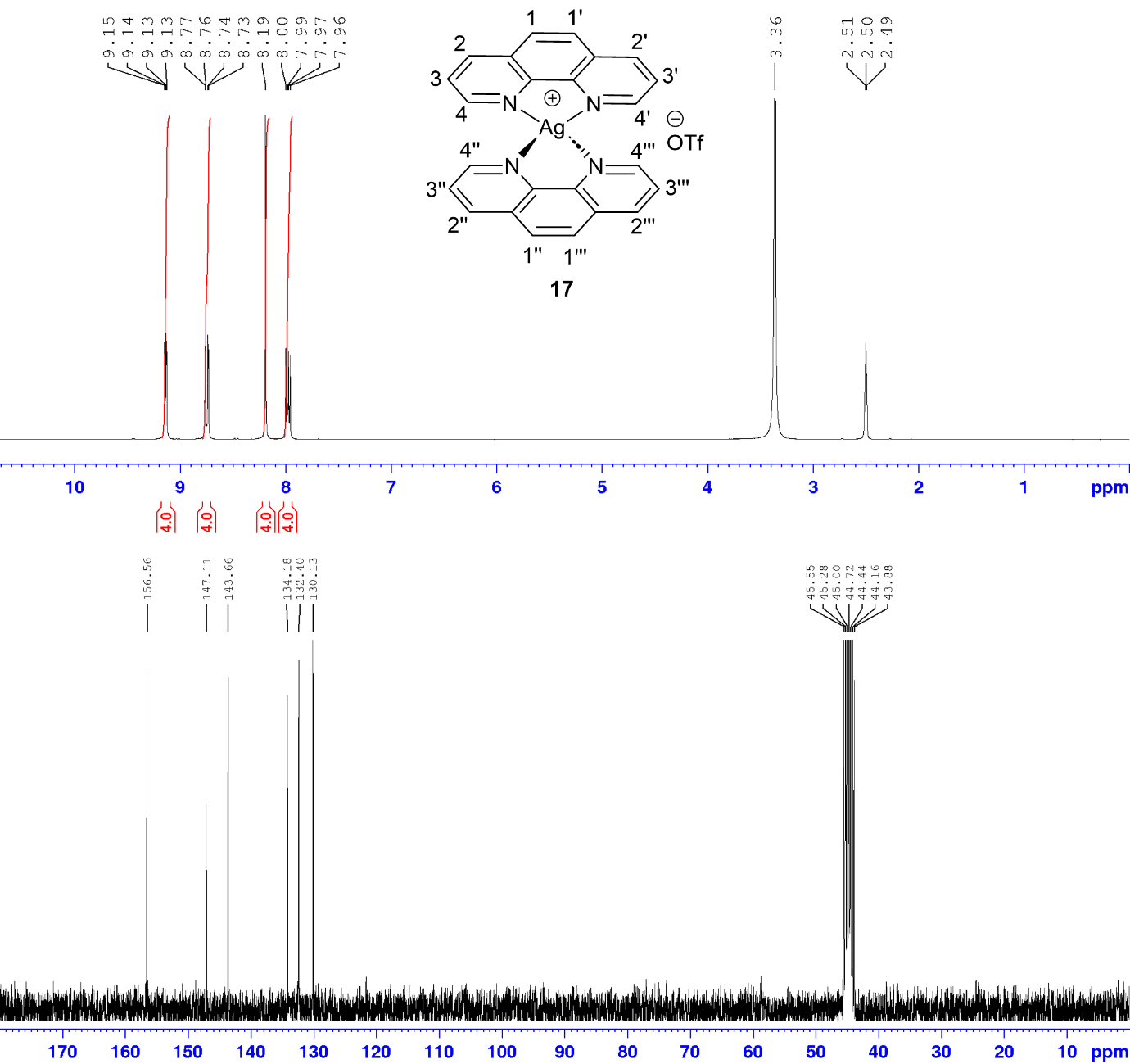


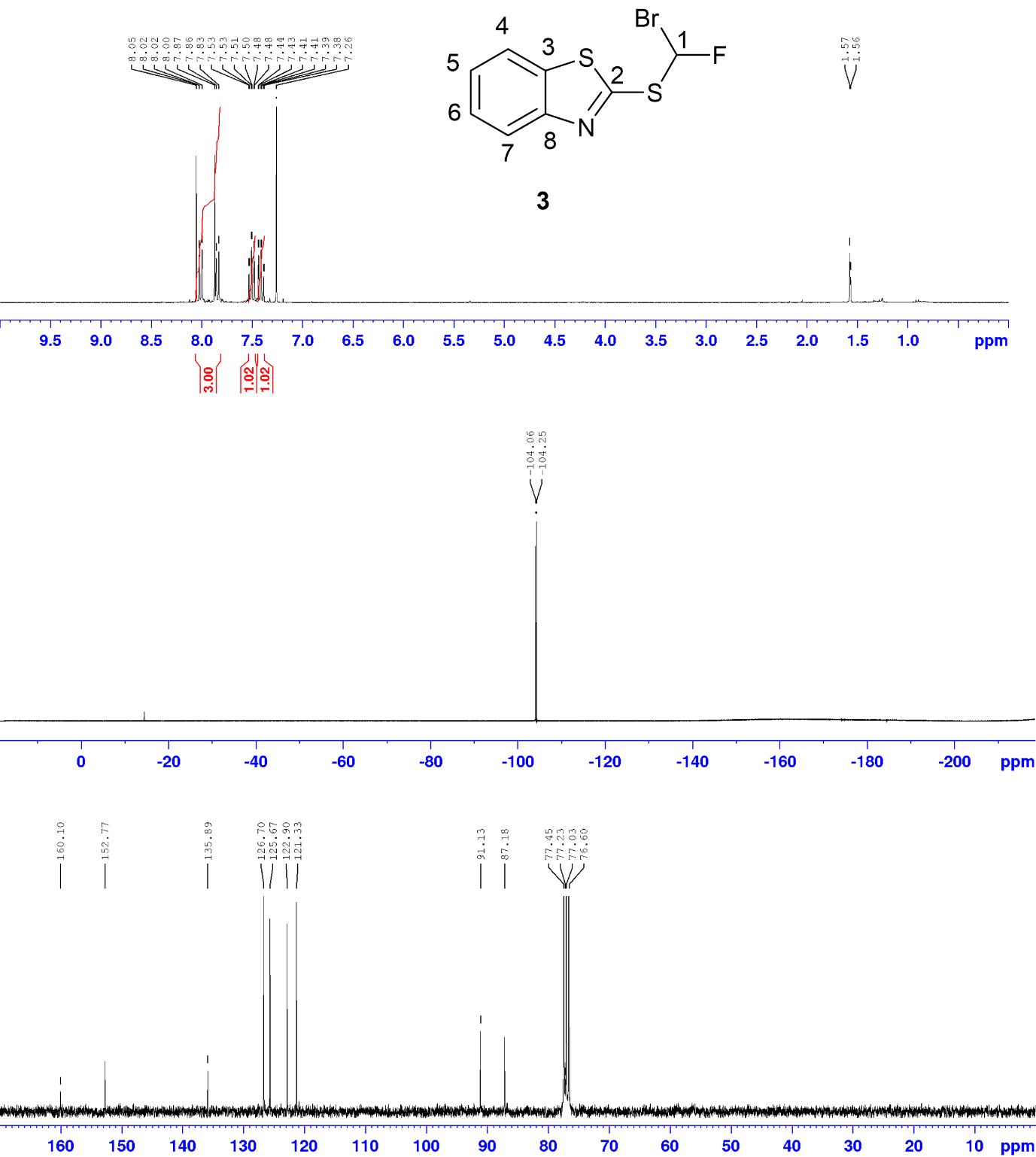


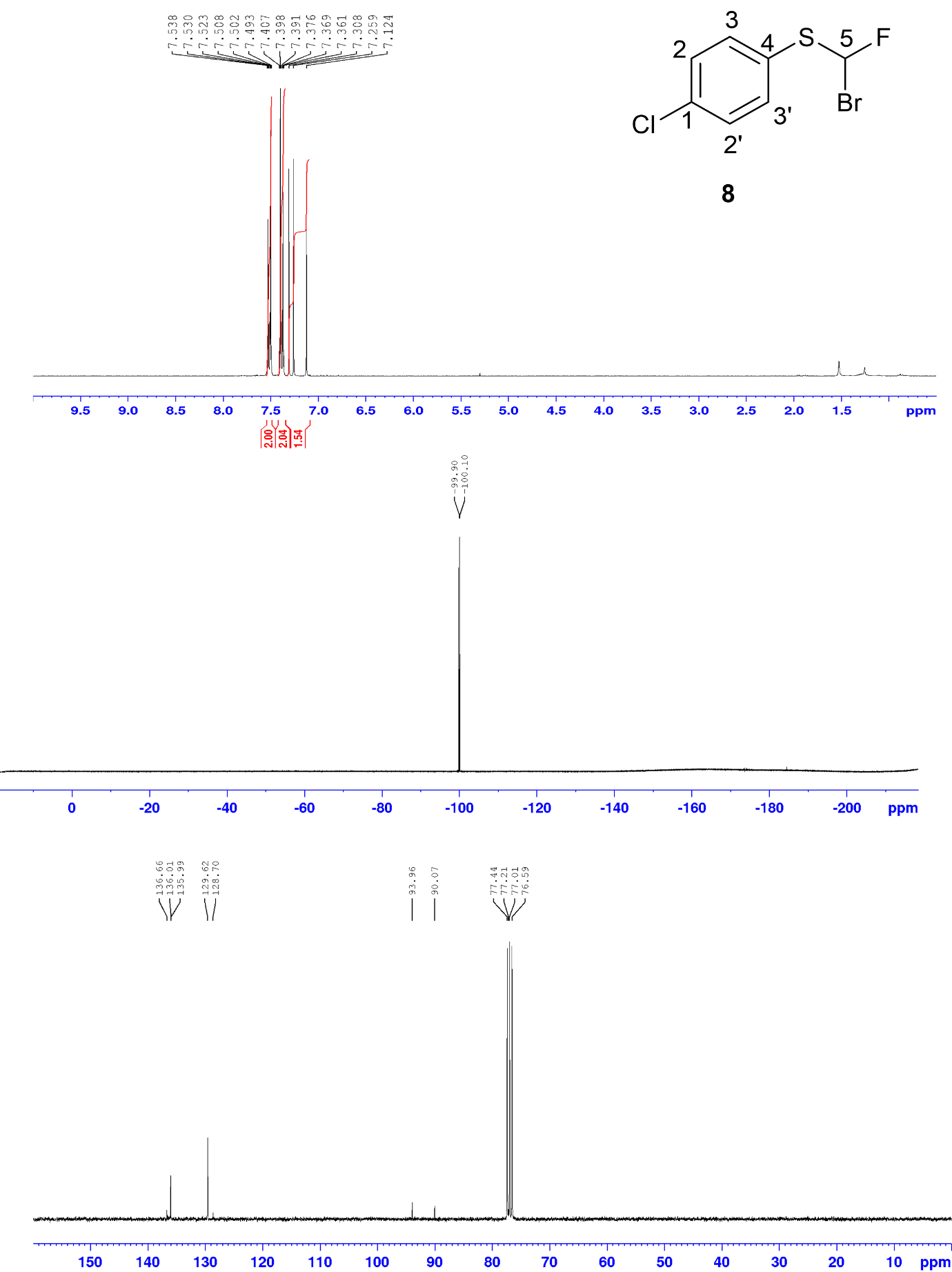


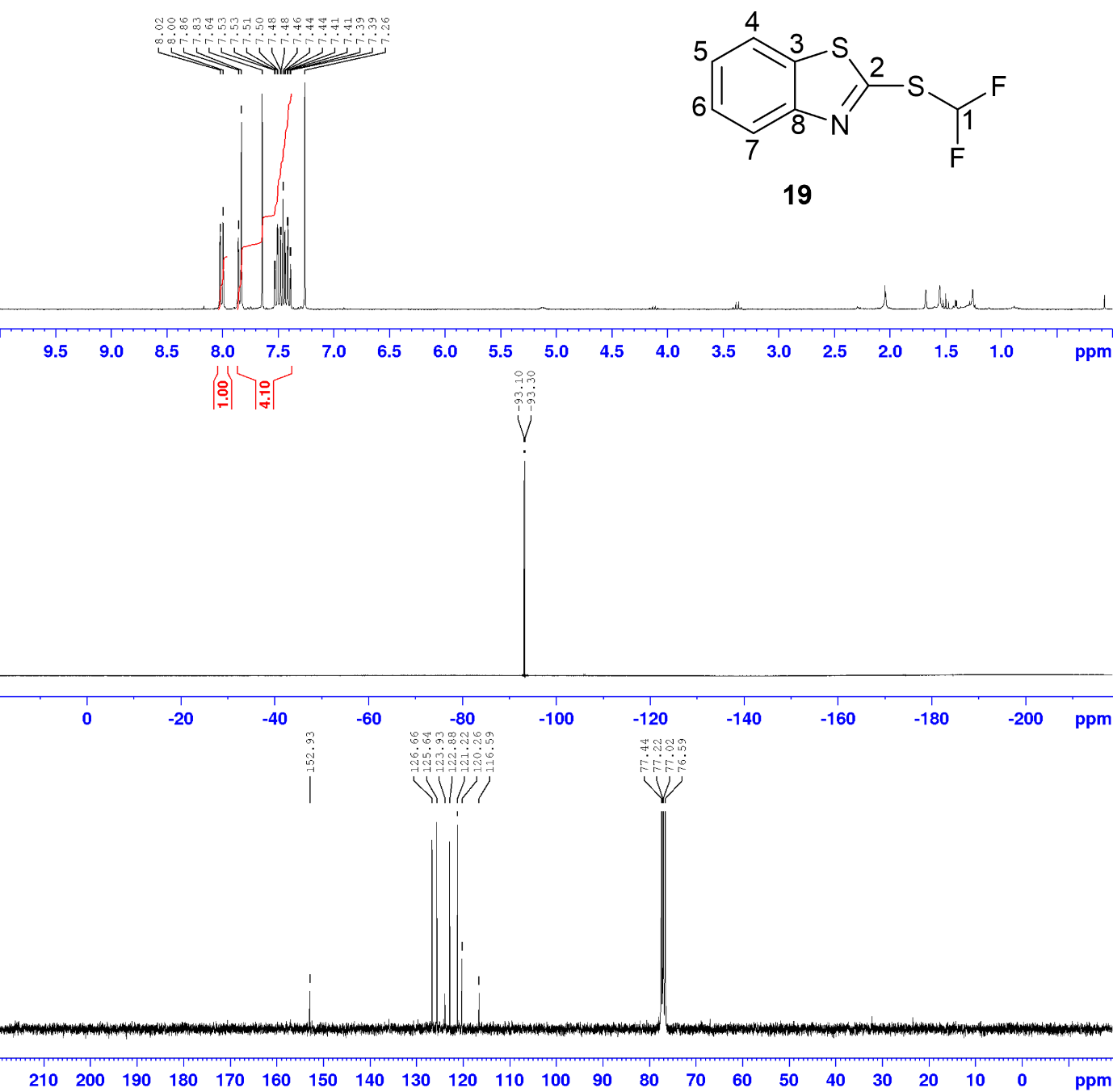


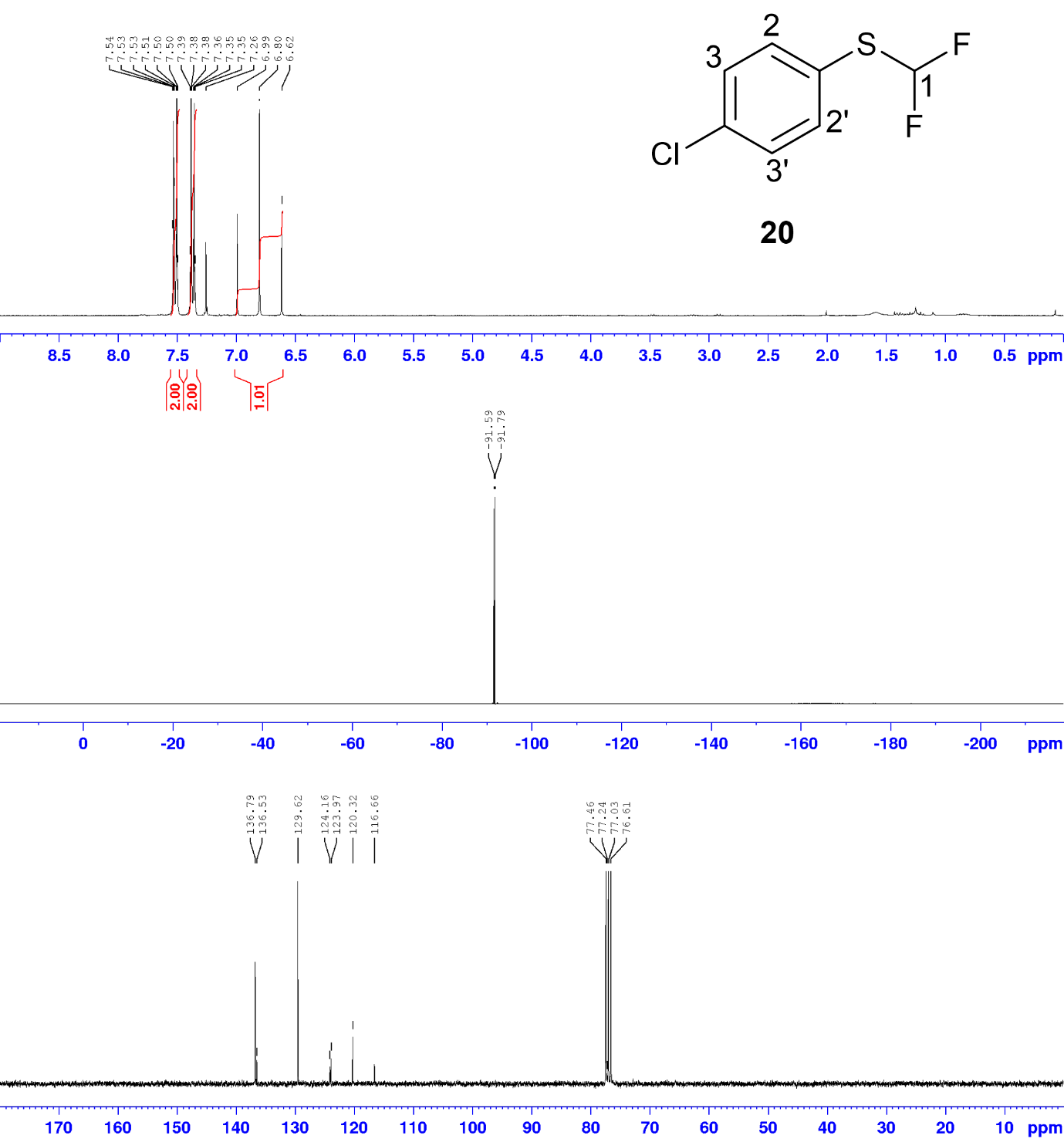


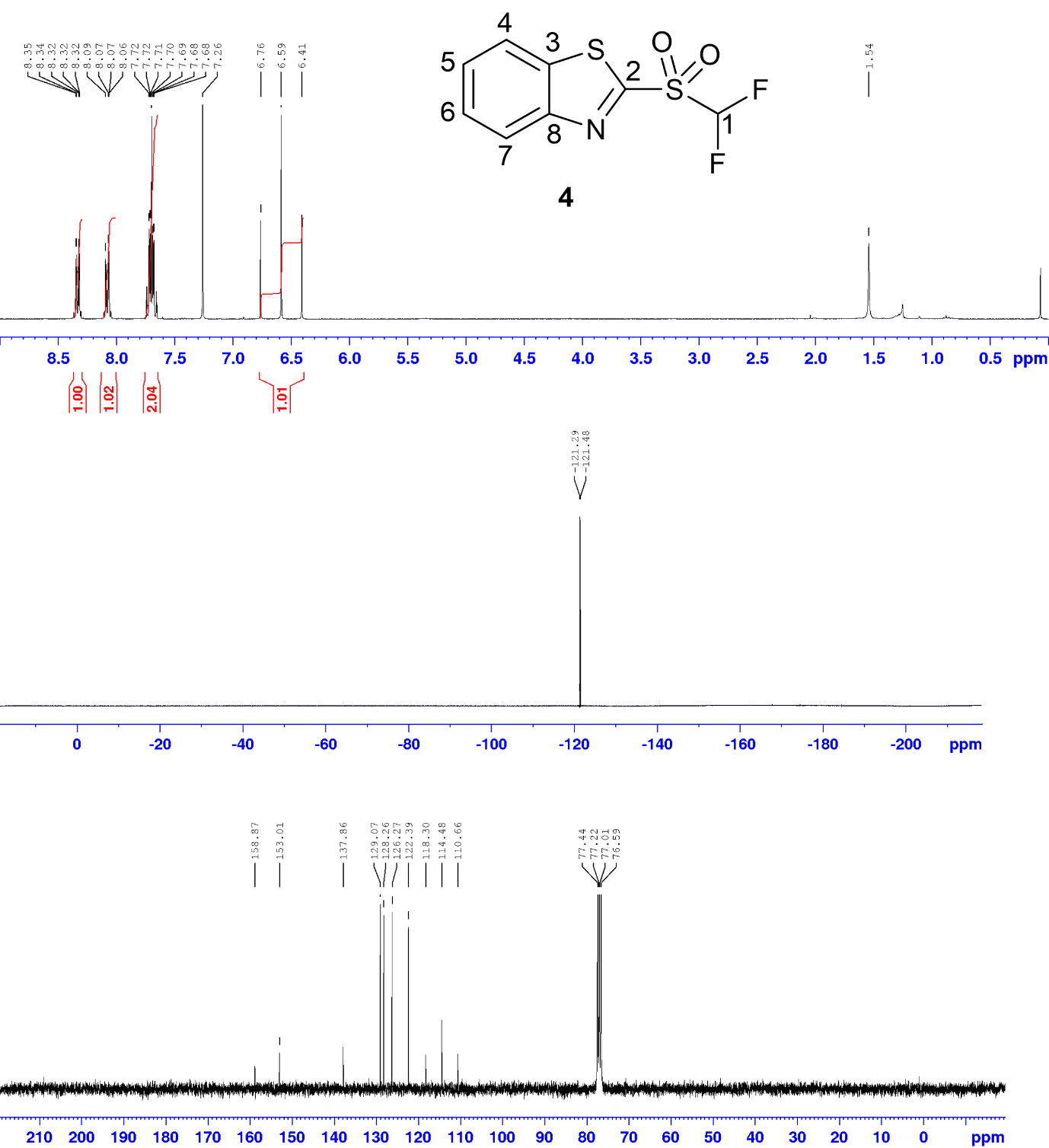


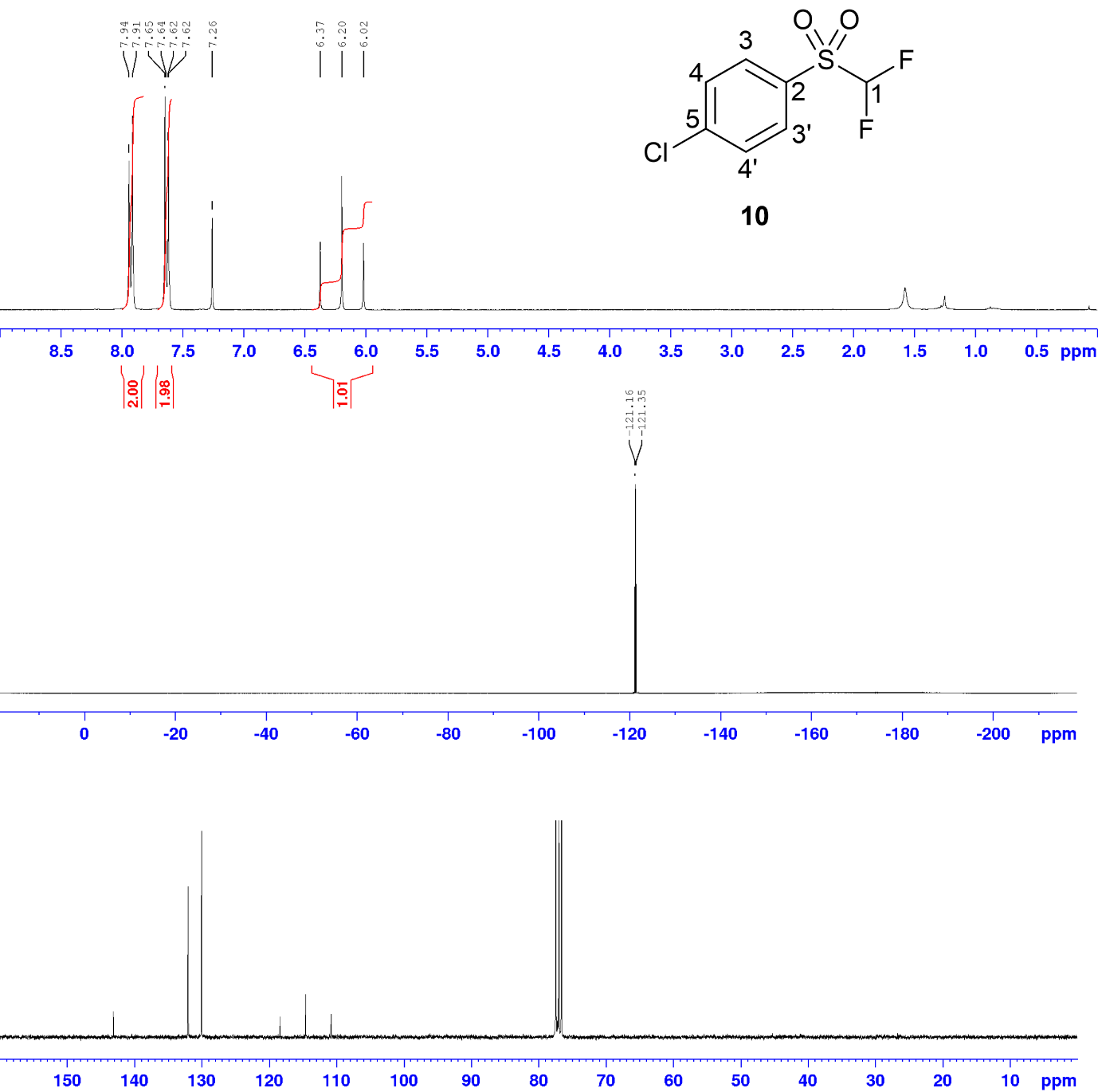


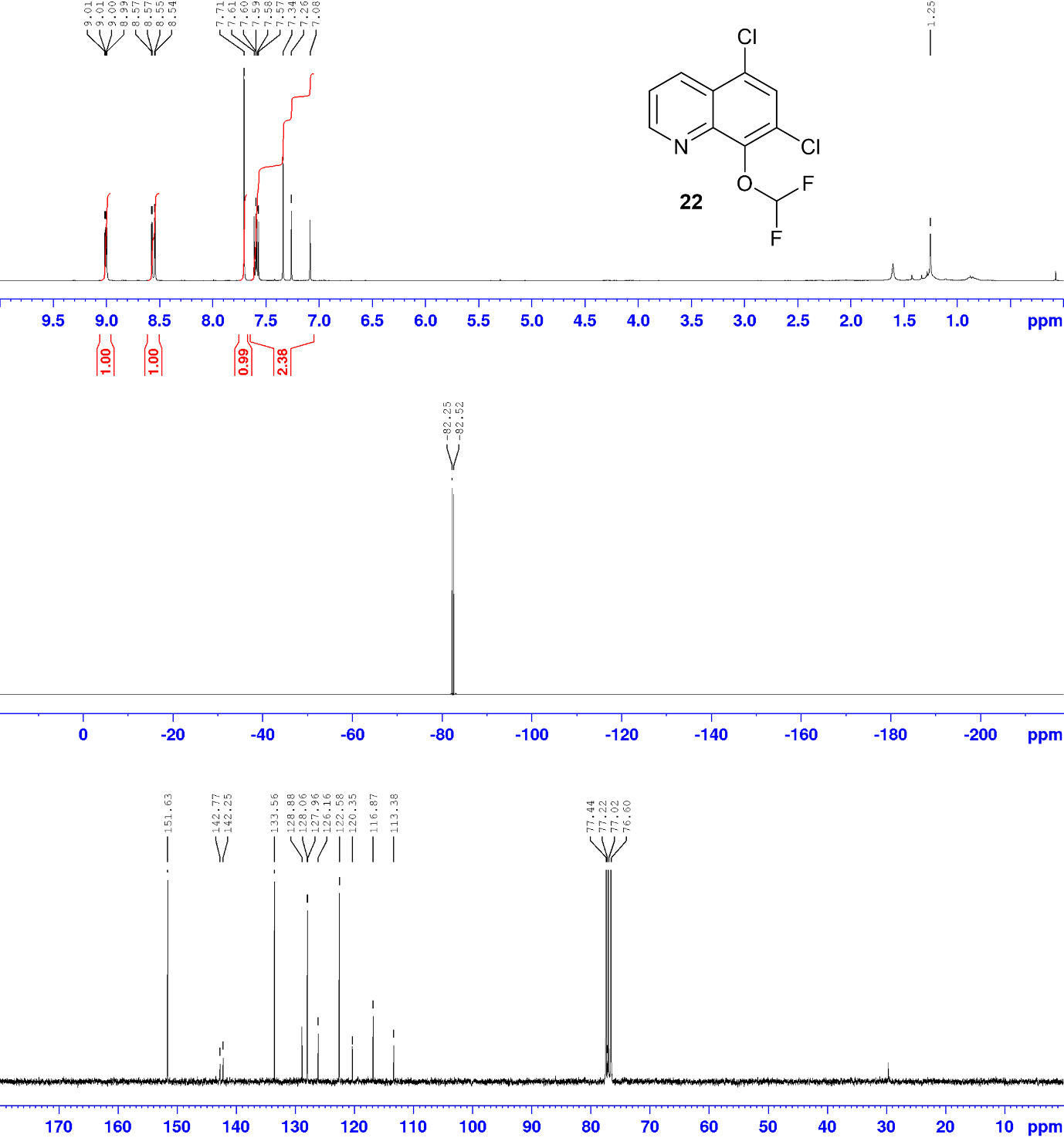


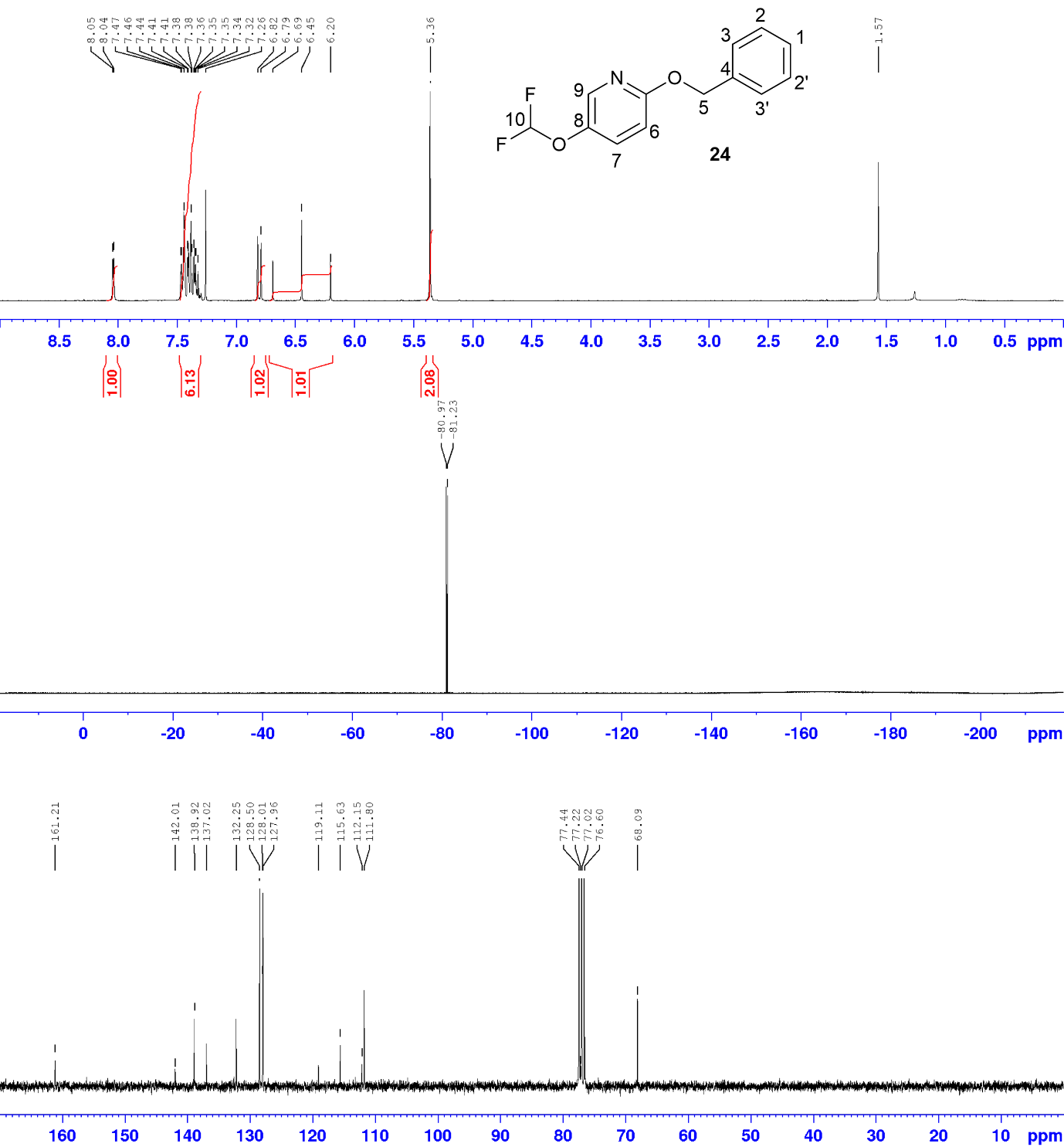


# X-ray Crystallography Data

Single-crystal X-ray data were collected on a Bruker D8-QUEST diffractometer, equipped with an Incoatec IμS Cu microsource (λ = 1.5418 Å) and a PHOTON-III detector operating in shutterless mode. The crystal temperature was held at 180(2) K using an Oxford Cryosystems open-flow N_2_ Cryostream. The control and processing software was Bruker *APEX4* (ver. 2021.4-0). Structures were solved using *SHELXT*^3^ and refined using *SHELXL*.^4^ All non-H atoms were refined with anisotropic displacement parameters. H atoms on C atoms were placed geometrically and refined as riding. The H atom of the carboxylic acid group in **15** was located and refined freely with an isotropic displacement parameter. For **10**, the non-centrosymmetric structure was refined explicitly as an inversion twin.

The crystal structures have been deposited at the Cambridge Crystallographic Data Centre: deposition numbers 2421950, 2421951, 2421952. The structures can be retrieved from: <https://www.ccdc.cam.ac.uk/structures/>

**Molecular structure of 4 with displacement parameters at 50% probability for non-H atoms:**


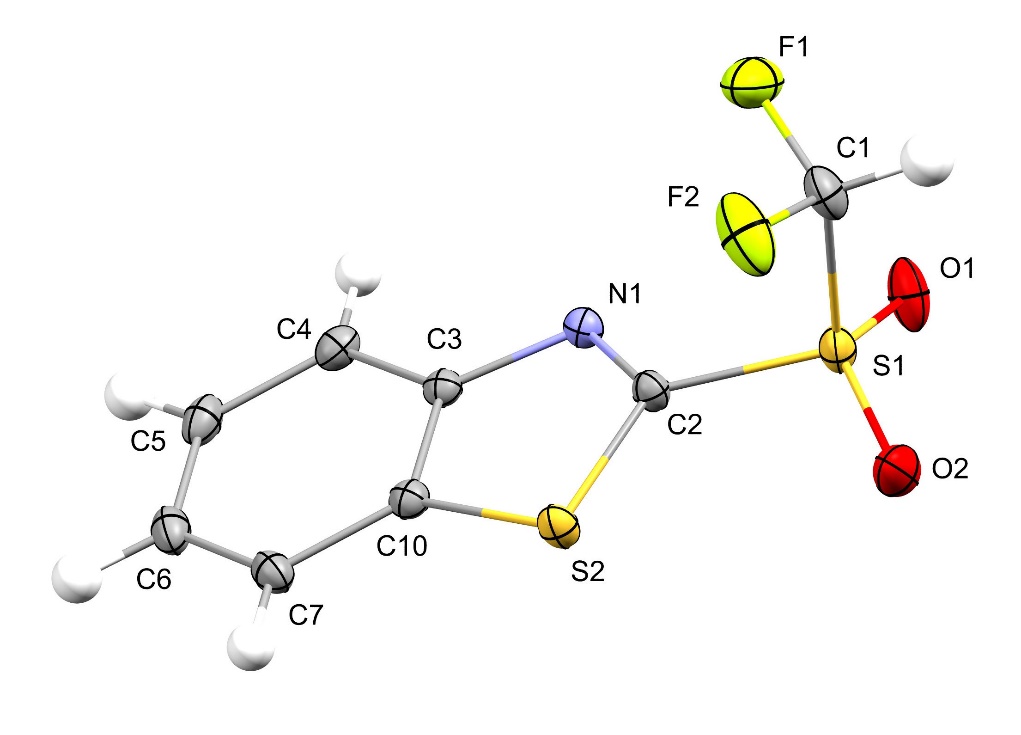


**Molecular structure of 10 with displacement parameters at 50% probability for non-H atoms (two molecules in the crystallographic asymmetric unit):**


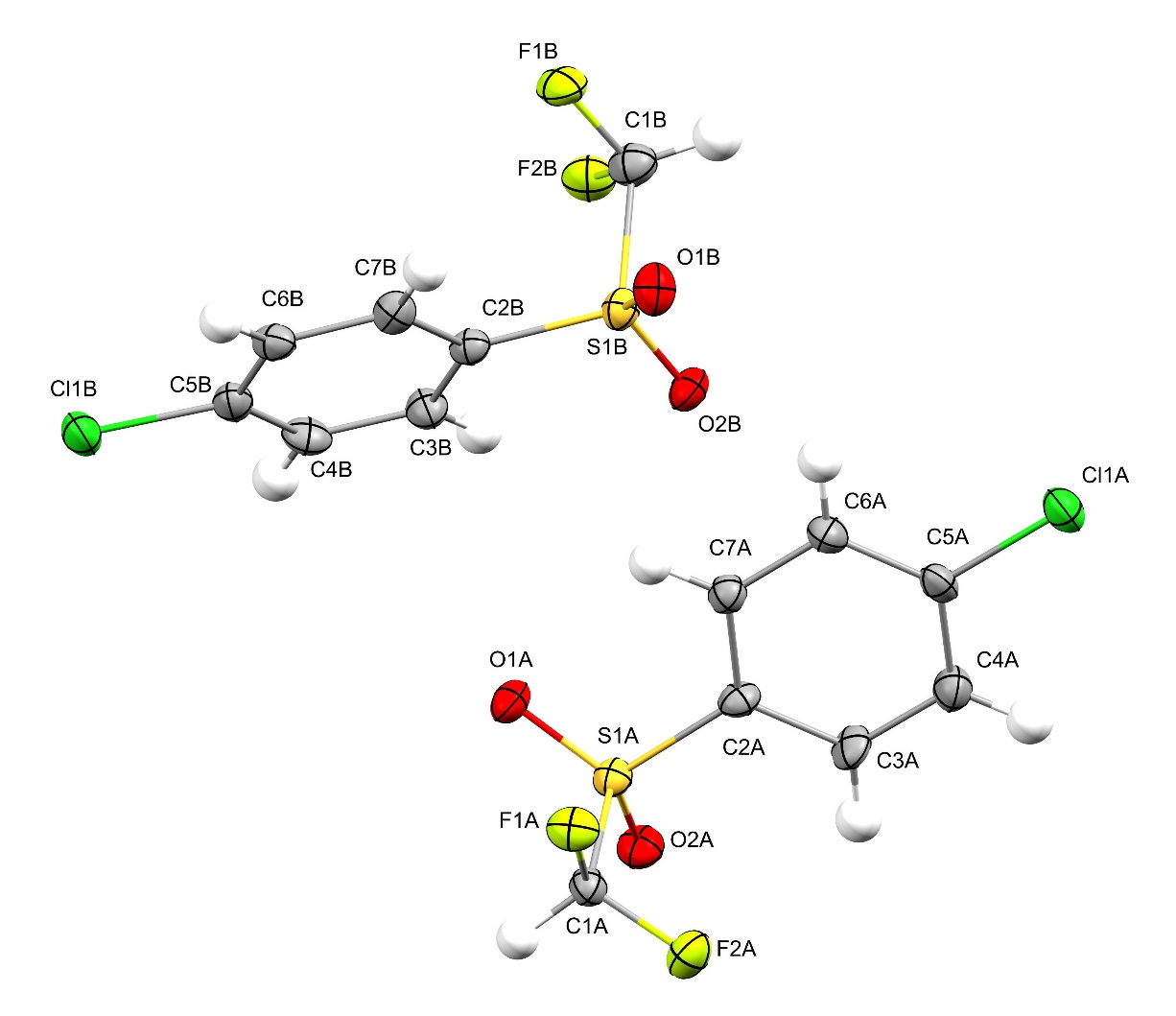


**Molecular structure of 15 with displacement parameters at 50% probability for non-H atoms:**


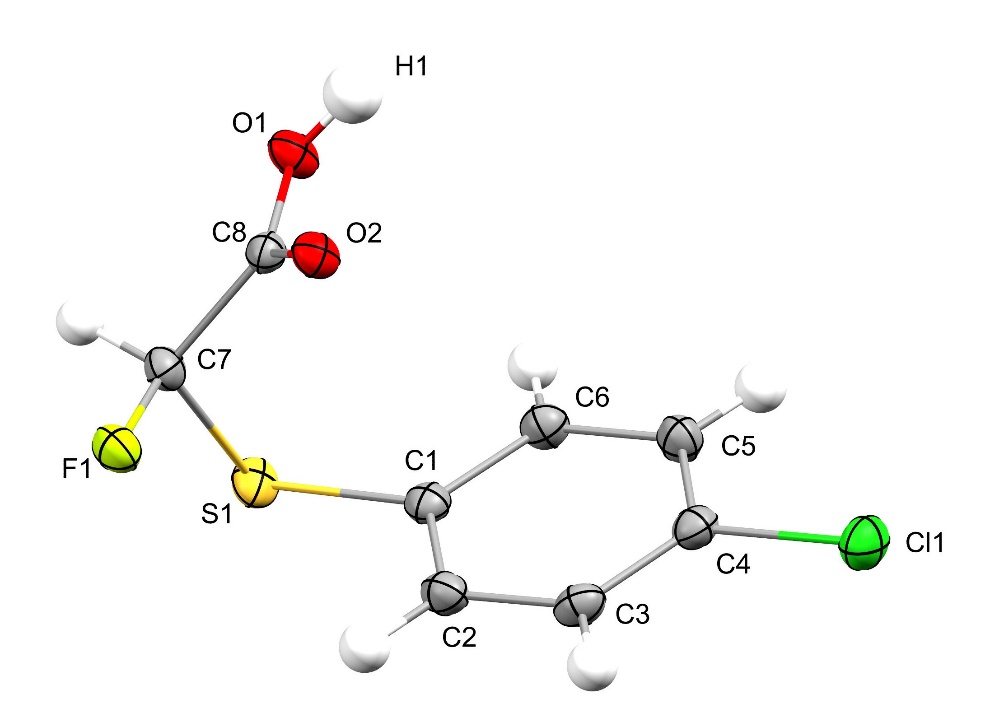


**Summary of crystal and refinement data**

|  | **4** | **10** | **15** |
| --- | --- | --- | --- |
| CCDC number | 2421951 | 2421952 | 2421950 |
| Cambridge data no. | AB_B1_0082 | AB_B1_0083 | AB_B1_0092 |
| Chemical formula | C_8_H_5_F_2_NO_2_S_2_ | C_7_H_5_ClF_2_O_2_S | C_8_H_6_ClFO_2_S |
| Formula weight | 249.25 | 226.62 | 220.64 |
| Temperature / K | 180(2) | 180(2) | 180(2) |
| Crystal system | monoclinic | orthorhombic | triclinic |
| Space group | *P* 2_1_/*n* | *P c a* 2_1_ | *P* –1 |
| a / Å | 6.7746(2) | 11.1961(10) | 7.0993(2) |
| b / Å | 8.3268(3) | 7.7034(8) | 7.5579(2) |
| c / Å | 16.8787(6) | 20.1877(17) | 8.9910(3) |
| alpha / ° | 90 | 90 | 90.3426(15) |
| beta / ° | 91.541(2) | 90 | 105.9557(14) |
| gamma / ° | 90 | 90 | 92.9779(14) |
| Unit-cell volume / Å ^3^ | 951.80(6) | 1741.1(3) | 463.10(2) |
| Z | 4 | 8 | 2 |
| Calc. density / g cm^–3^ | 1.739 | 1.729 | 1.582 |
| F(000) | 504 | 912 | 224 |
| Radiation type | Cu Kα | Cu Kα | Cu Kα |
| Absorption coefficient / mm^–1^ | 5.228 | 6.178 | 5.631 |
| Crystal size / mm^3^ | 0.20 x 0.14 x 0.04 | 0.10 x 0.06 x 0.02 | 0.14 x 0.12 x 0.10 |
| 2-Theta range / ° | 10.5–136.2 | 8.8–136.7 | 10.2–136.4 |
| Completeness to max 2 | 0.998 | 0.999 | 0.982 |
| No. of reflections measured | 13461 | 15027 | 6391 |
| No. of independent reflections | 1724 | 3157 | 1662 |
| *R*(int) | 0.0422 | 0.1130 | 0.0341 |
| No. parameters / restraints | 136 / 0 | 236 / 1 | 122 / 0 |
| Final *R*1 values (*I* > 2σ(*I*)) | 0.0265 | 0.0830 | 0.0275 |
| Final *wR*(F^2^) values (all data) | 0.0688 | 0.2375 | 0.0742 |
| Goodness-of-fit on *F*^2^ | 1.063 | 1.071 | 1.064 |
| Largest difference peak & hole / e Å^–3^ | 0.307, –0.284 | 0.525, –0.558 | 0.221, –0.219 |
| Flack parameter |  | 0.19(8) |  |

# Supplementary References

1. Kirihara, M. *et al.* Synthesis of Monofluoromethylcyclopropanes from Alkenes without Using Freons: Novel Synthesis of Chlorofluoromethyl Phenyl Sulfide and Its Application in Cyclopropanation. *Chem. Lett.* **42**, 1377–1379 (2013).

2. Sap, J. B. I. *et al.* [18F]Difluorocarbene for positron emission tomography. *Nature* **606**, 102–108 (2022).

3. Sheldrick, G. M. *SHELXT* – Integrated Space-group and Crystal-structure Determination. *Acta Cryst. Sect. A,* **71**, 3–8 (2015).

4. Sheldrick, G. M. Crystal Structure Refinement with *SHELXL*. *Acta Cryst. Sect. C,* **71**, 3–8 (2015).
